# Supplementary material for: A prospective single-masked, non-inferiority, parallel-group randomized controlled trial of the efficacy of a ChatGPT-based AI chatbot to improve Boston bowel preparation scores for colonoscopy preparation: A trial protocol
Source: PLoS One. 2025 Oct 15;20(10):e0334349. doi: 10.1371/journal.pone.0334349 (PMC12527164; doi:10.1371/journal.pone.0334349)
Supplement: S1 File — (PDF) [file pone.0334349.s002.pdf]

FULL TRIAL PROTOCOL TITLE

PROSPECTIVE SINGLE BLINDED RANDOMIZED CONTROLLED TRIAL ON THE EFFECTIVENESS OF USING ARTIFICIAL INTELLIGENCE (AI) CHATBOT TO IMPROVE BOSTON BOWEL PREPARATION SCORES (BPPS) FOR COLONOSCOPY PREPARATION

VERSION 2

DATE: 10/03/2025

SUPERVISOR

DR. (MR.) NABIL MOHAMMAD AZMI

STATISTICIANS

MUHAMMAD IRFAN ABDUL JALAL (Lead Statistician and Trial Methodologist)

DR. YEW SHENG QIAN (Statistician)

NAME	MATRIC NUMBER
SITI HAMIZAH BINTI MOHD ASHAR	A188116
MUHAMMAD IRFAN BIN MOHD NAZRI	A189561
YOUNG JIE	A181782
NAGULAN A/L GANESON	A188393
JOANE K AUGUSTINE	A188351

Introduction and Literature Review

Colonoscopy is a vital diagnostic tool for colorectal cancer and adenomas, significantly reducing morbidity and mortality through early detection **(1)**. However, its effectiveness relies heavily on high-quality bowel preparation, which impacts adenoma detection rates (ADR) and procedural success. Inadequate preparation decreases colonoscopy efficiency due to the necessity for repeat procedures, generating increased expenses **(2)**. Furthermore, it engenders delays in diagnosing malignant or precancerous lesions, curtails the adenoma detection rate (ADR), and increases procedural times and possibly patient risk. However, the prevalence of suboptimal colonoscopies across endoscopy units evinces considerable variability in studies, spanning from 6.8% to 33%. **(3)** A multitude of factors have been linked to inadequate bowel preparation, prompting endeavors to mitigate suboptimal bowel cleansing through interventional studies aimed at high-risk patients with poor bowel cleansing

Patient compliance with these pre-procedural instructions is crucial for achieving high-quality results and reducing the likelihood of complications or the need for repeat procedures. Despite the apparent importance of bowel preparation, ensuring patient adherence to pre-procedural instructions remains a significant challenge. Several barriers to adequate bowel preparation exist, including non-compliance, lack of understanding of instructions, and anxiety regarding the procedure. Sociodemographic factors, such as age and education level, have been identified as predictors of inadequate preparation **(4).**

Confronted with this challenge, the incorporation of Artificial Intelligence (AI) technology presents an innovative approach to bowel preparation scoring. Particularly noteworthy are the significant achievements of Convolutional Neural Networks (CNN) in medical image analysis, exemplified by the AI system ENDOANGEL, which has brought a higher level of accuracy and consistency to bowel preparation scoring **(5)**. These advancements suggest that deep learning and AI could assist clinicians in improving the accuracy and consistency of bowel preparation scoring. In the past few years, CNNs have advanced in bowel preparation assessment. However, they fall short in clinical usability and human-centered communication. In other words, most deep learning models have this rigid boundary in a discriminative way instead of parametrically assigning probabilities to work together with clinicians. Conversely, ChatGPT, a large language processing model, has shown proficiency in processing medical texts and enhancing doctor-patient communication, exemplified by its ability to provide contextually informed suggestions on colonoscopy follow-up timings **(6)**. Furthermore, ChatGPT 4.0 has shown potential in image recognition, enhancing its multimodal analysis capabilities.

Artificial intelligence has become a transformative force in healthcare, with applications ranging from clinical decision support systems to patient management tools. Among these innovations, AI chatbots stand out due to their ability to simulate human conversation, provide tailored

information, and engage with patients interactively. Use of AI chatbots is able to address current health care challenges, such as shortages of healthcare providers, which reduce the availability and accessibility of health care services **(7-9)**. AI chatbots use artificial intelligence (AI), including machine learning (a statistical means of training models with data so that they can make predictions based on a variety of features) and natural language processing (NLP; the ability to recognize and analyze verbal and written language) to interact with humans via speech, text, or other inputs and outputs on mobile, web-based, or audio-based platforms **(10,11)**. Many of these agents are designed to use NLP so that users can speak or write to the agent as they would to a human. The agent can then analyze the input and respond appropriately in a conversational manner.

AI chatbots have been developed for many different aspects of the health sector to support healthcare professionals and the general public. Specific uses include screening for health conditions, triage, counseling, at-home health management support, and training for healthcare professionals **(12)**. With phone, mobile, and online platforms being widely accessible, conversational agents can support populations with limited access to health care or poor health literacy **(13,14)**. They also have the potential to be affordably scaled up to reach large proportions of a population **(15)**. Due to this accessibility, conversational agents are also a promising tool for the advancement of patient-centered care and can support users' involvement in the management of their own health **(14,16)**. Personalizable features have the potential to improve usability and satisfaction further, although more research is needed to evaluate their effectiveness in achieving their stated health outcomes and reducing costs and to ensure that there are no negative consequences for decision making or privacy. These systems use NLP to understand and respond to patients' questions conversationally, thereby overcoming one of the most significant barriers to effective communication: the complexity of medical information. In the context of colonoscopy preparation, AI chatbots can be designed to provide specific instructions on dietary restrictions, bowel cleansing, and medications, ensuring that the patient receives accurate, easy-to-understand guidance.

Based on research, The Effectiveness of Artificial Intelligence Conversational Agents in Health Care: Systematic Review, conducted by Madison Milne-Ivs in 2020 **(17)**, 31 studies have evaluated the use of AI chatbots for educational purposes in healthcare. The study designs varied widely, with 29% (9/31) using cross-sectional designs, 26% (8/31) using RCTs, 23% (7/31) using qualitative methods, 19% (6/31) using cohort studies, and 1% using a cluster crossover design.

There was a wide variety of areas of health care targeted by the conversational agents of the included studies **(17)**. The most significant proportion of them (12/31, 39%) addressed mental health issues [13,32-42], with 19% (6/31) providing some form of clinical decision or triage support [8,12,40,42-44] and treatment support (including encouraging users to get screened) , 10% (3/31) being used to support training of health care students and the screening or diagnosis of users

[14,38,51], 7% (2/31) targeting physical health and layperson medical education; 1 agent was designed to help monitor users' speech.

Overall, about three-quarters of the studies (22/30, 73%) reported positive or mixed results for most of the outcomes. Perceived ease of use or usefulness (27/30, 90%), the process service delivery or performance (26/30, 87%), appropriateness (24/30, 80%), and satisfaction (26/31, 84%) were the outcomes that had the most support from the studies. Just over three-quarters (23/30, 77%) of the studies also reported positive or mixed evidence of effectiveness. However, very few studies discussed the cost-effectiveness (5/30, 17%, coded as positive or mixed) or safety, privacy, and security (14/30, 47%, coded as positive or mixed) outcomes for the agents being evaluated. About a quarter of studies (8/30, 27%) had neither positive nor mixed reported evidence for more than half of the SF/HIT (synthesis framework for the assessment of health information technology) outcomes. **(17)**

A systematic review highlighted that conversational agents generally improve patient engagement and health outcomes, with 75% of studies reporting positive results in usability and effectiveness. Specifically, they enhance adherence to pre-procedure protocols through interactive guidance. In addition to providing information, AI chatbots can help alleviate patient anxiety, which is another common barrier to successful colonoscopy preparation. Anxiety around the procedure, the bowel prep process, and potential discomfort can discourage patients from completing their preparation effectively. AI chatbots, by offering empathetic responses and providing reassurance, can help ease these concerns and improve patient compliance.

The ultimate goal of using AI chatbots for patient preparation is to improve clinical outcomes. In the case of colonoscopy **(18)**, clinical outcomes can be measured by the quality of bowel preparation, which directly affects the diagnostic efficacy of the procedure, as measured by the Boston Bowel Preparation Scale (BBPS) score (total score range, 0-9, where 0 indicates extremely unsatisfactory bowel preparation and 9 indicates complete bowel preparation), evaluated by the endoscopist during the procedure, who scored from 0 to 3 for the three broad regions of the colon: right, transverse, and left (the cecum and ascending colon, the hepatic and splenic flexures, and the descending colon, sigmoid colon, and rectum). If the mucosa could not be visualized because of remaining stool or food residue, this segment would receive a score of 0; a score of 3 means that the whole mucosa of the colon segment can be seen clearly without residual staining, small fragments of stool, or opaque liquid. The endoscopist was blinded to the group of patients. High-quality bowel preparation reduces the likelihood of repeat colonoscopies, increases the chances of detecting abnormalities, and reduces the risk of complications.

Secondary outcomes included the rate of adequate preparation (a score of 2 for all regions), the polyp detection rate, the biopsy-verified adenoma detection rate, and the cecal intubation rate. A questionnaire can be used to evaluate patient compliance with bowel preparation (rate of compliance with diet restriction and laxative use). A self-rated score from 0 to 10 points was used

in the questionnaire to assess pre procedure anxiety (measured by self-rated sleep quality before the procedure [where 0 indicates inferior sleep quality and 10 indicates sleep quality that is the same as usual]), overall satisfaction with bowel preparation (where 0 indicates an inferior experience with a very painful process and many adverse effects and 10 indicates the examination has no adverse effects), and willingness to undergo another colonoscopy if indicated (where 0 indicates great reluctance to undergo the examination again and 10 indicates willingness to undergo the examination again if necessary)

While AI chatbots show great promise, there are several challenges and limitations to their use in healthcare. One of the key challenges is the technological barrier, particularly in the complexity of natural language understanding **(17)**. However, research by Marni H. Wilkoff on Can Artificial Intelligence Create an Accurate Colonoscopy Bowel Preparation Prompt, in 2024, suggested that providing ChatGPT with contextualized medical information can help further expand on areas that are considered vague. In addition to utilizing ChatGPT to incorporate contextual knowledge, an experienced gastroenterologist should review and provide additional expertise as well. Chatbots must be able to process and respond to a wide variety of patient queries, which can be difficult when patients ask nuanced or unanticipated questions. Additionally, the chatbot's performance is heavily dependent on the quality of the underlying algorithms and data. Poor design or insufficient data can lead to errors or unhelpful responses, undermining the effectiveness of the tool. Other chatbots-specific negative feedback was that the virtual doctor did not have the ability to go deep enough or provide access to different materials, that too much information was provided, or the interaction was too long, the use of nonverbal expressions by the avatar, and a lack of clarity regarding the aim of the chatbot. Some students who used the virtual patients also reported that it was difficult to empathize and that the agent did not sufficiently encompass real situational complexity. Based on research conducted by Marni H. Wilkoff on Can Artificial Intelligence Create an Accurate Colonoscopy Bowel Preparation Prompt? In 2024, to ensure the prompt is appropriate for various education levels, it is crucial to modify it, allowing all patients to understand the instructions. Having instructions that are too advanced for an individual can lead to poor bowel preparation compliance and success. Creating multiple versions of bowel preparation instructions has the potential to create more work for physicians, but with the help of ChatGPT, one can query the large language model to customize the response to a specific education level.

Another limitation is patient acceptance. Some patients may be hesitant to trust AI-based systems, especially when it comes to medical advice or procedural instructions. This is particularly true for older adults or individuals with lower levels of health literacy or technological proficiency. Ensuring that AI chatbots are designed to be user-friendly and accessible is crucial to overcoming this barrier. As for patients with technology illiteracy, it is essential to prepare the patient with hands-on guidance and offer them reassurance to build their confidence in using the AI chatbot effectively. Starting by providing basic education and demonstration, making sure patients have

easy access to the AI chatbots by bookmarking or installing apps, walking them through the steps and clarifying any concerns they have.

Traditional methods of patient preparation for colonoscopy often rely on printed materials, pamphlets, or phone calls from healthcare professionals. While effective to some extent, these methods lack interactivity and personalization. To address this, a designated laptop in the clinic can be used during the consent process, allowing patients to interact with an AI chatbot for colonoscopy preparation. This setup ensures immediate assistance if patients face difficulties, with prompts and guidance provided by staff. This hybrid approach combines the 24/7 availability of AI chatbots with direct support, enhancing patient understanding and adherence to preparation guidelines, ultimately improving outcomes like detection rates during colonoscopy.

Although the initial studies on AI chatbots in healthcare are promising, there is still a need for further research to understand their potential in pre-procedural patient education fully. Future studies should focus on improving the natural language processing capabilities of chatbots to ensure they can handle more complex patient interactions. Additionally, research is needed to evaluate the long-term impact of AI chatbot use on patient outcomes, such as the quality of colonoscopy preparation and procedural success. There is also a need to explore patient attitudes towards AI chatbots and identify strategies to increase acceptance and trust in these systems. Further, integration with electronic health records (EHRs) could enable AI chatbots to provide even more personalized and contextually aware guidance, improving their effectiveness and reducing the risk of errors.

AI chatbots represent a promising tool for enhancing patient preparation for colonoscopy. They offer several advantages, including personalized education, real-time support, and improved patient adherence to preparation guidelines. Early studies suggest that AI chatbots can lead to better clinical outcomes, such as improved bowel preparation. However, challenges such as technological limitations, patient trust, and acceptance need to be addressed. As AI technology continues to evolve, further research will be critical in refining chatbot systems and exploring their full potential in improving patient preparation for medical procedures like colonoscopy.

Problem Statement

Traditional pre-colonoscopy counseling requires significant time from healthcare workers to explain procedures, limiting efficiency and patient turnover. Inadequate bowel preparation exacerbates this issue, leading to repeat procedures and increased costs. However, no study has yet evaluated the effectiveness of AI in improving the Boston Bowel Preparation Scale (BBPS) for colonoscopy preparation. By addressing this gap, AI chatbots could provide personalized guidance, reduce healthcare worker burden, improve preparation quality, and enhance patient throughput.

General objectives

To determine the effectiveness of artificial intelligence (AI) chatbot in improving bowel preparation among colonoscopy patients in Hospital Tuanku Mukhriz (HCTM), compared to conventional instructions

Specific Objectives

1. To determine the effectiveness of AI chat bot in relieving anxiety among colonoscopy patients in HCTM, compared to conventional instructions.
2. To determine the effectiveness of AI chat bot in improving satisfaction among colonoscopy patients in HCTM, compared to conventional instructions.

Hypothesis

Artificial intelligence (AI) chat bot can improve bowel preparation, anxiety level and patient's satisfaction among colonoscopy patients in Hospital Tuanku Mukhriz (HCTM), compared to conventional instructions

Null hypothesis

Artificial intelligence (AI) chat bot cannot improve bowel preparation, anxiety level and patient's satisfaction among colonoscopy patients in Hospital Tuanku Mukhriz (HCTM), compared to conventional instructions

Study Design

This is a prospective, single-centre, single-masked (outcome-assessor), two-parallel arm, superiority randomized controlled trial. The primary goal is to assess whether AI chatbot counseling improves bowel preparation quality (BBPS) for elective colonoscopy. Eligible patients will be randomized (block randomization, block size = 4) in a 1:1 ratio to either the AI intervention group or the control group receiving standard instructions. Endoscopists, who will score the BBPS during colonoscopy, will be masked to group allocation. The protocol adheres to SPIRIT 2025 guidelines and has received ethical approval from the UKM Research Ethics Committee (Ref: UKM PPI/111/18, Date of approval: 14th Mar 2025). Trial registration with ClinicalTrials.gov has been approved on 01/04/2025 (ID: NCT06905782).

Table 1 shows the SPIRIT 2025 schedule of participant enrollment, administration of interventions and evaluations of trial endpoints. The structured, summarized details of the trial design, based on

the World Health Organization (WHO) Trial Registration Dataset, are given in Table 2. The planned participant data collection is summarized in Table 3. Written informed consent will be obtained from each study participant by the trained study recruiters.

Table 1. Schedule of participant enrollment, administration of interventions and assessments of study outcomes for this trial (adapted from SPIRIT 2025 guideline).

	**Trial Period**				
	**Enrollment**		**Post Randomization**		**During colonoscopy**
**Time points**	$-t_i$ to 0	0	t_1	t_2 (after intervention)	t_2
**Enrollment**					
Screening for trial eligibility	X				
Informed consent	X				
Baseline participant demographic data collection	X				
Randomization		X			
**Interventions**					
ChatGPT-delivered instructions for					

colonoscopy preparation (Intervention of interest)			X		
Standard instructions for colonoscopy preparation (Comparator)			X		
**Assessments**					
Boston Bowel Preparation Scores (Bowel Preparation Quality)					X
DASS-21 score (Anxiety)				X	
PSQ-18 score<br><br>(Patient's satisfaction)				X	
Satisfactory bowel preparation status (Bowel Prep Scores ≥2 in all three colon segments)					X
Colonoscopy quality metrics					X

Table 2. Components of the WHO Registration Data Set. Extracted from the ClinicalTrials.gov registry and adapted from the SPIRIT 2025 guideline.

Components	Details
**Primary registry and trial identifying number**	Clinicaltrials.gov registry<br>NCT06905782<br>Weblink: https://clinicaltrials.gov/study/NCT06905782?id=NCT06905782
**Date of registration in the ClinicalTrials.gov registry**	01-04-2025
**Secondary Identifying Number**	JEP-2025-035
**Universal Trial Number (UTN)**	N/A
**Source of monetary and material support**	Faculty of Medicine, Universiti Kebangsaan Malaysia (UKM)
**Primary Sponsor**	Faculty of Medicine,

	Hospital Canselor Tuanku Muhriz (HCTM),<br>Universiti Kebangsaan Malaysia (UKM),<br>Jalan Yaacob Latif, Bandar Tun Razak,<br>56000, Cheras,<br>Kuala Lumpur, Malaysia.<br>Email: sepukm@ukm.edu.my
**Secondary Sponsor**	N/A
**Contact for Public Queries**	Dr Nabil Mohammad Azmi<br>Email: nabil@ukm.edu.my
**Contact for Scientific Queries**	Dr Nabil Mohammad Azmi<br>Email: nabil@ukm.edu.my
**Public Title**	ChatGPT for Bowel Preparation Counselling Before Colonoscopy
**Scientific Title**	Prospective Single-Blinded Randomized Control Trial on the Effectiveness of Using Large Language Model Artificial Intelligence Chatbot to Improve Boston Bowel Preparation Score (BBPS) for Colonoscopy Preparation
**Countries of Recruitment**	Malaysia
**Health Condition**	Suspected colorectal adenoma and colorectal cancer
**Interventions**	**Experimental Arm:** ChatGPT 4.0 Large Language Model (LLM)-aided Colonoscopy Counselling

	Randomized participants will be interacting with ChatGPT 4.0 for bowel preparation counselling prior to colonoscopy.<br><br>**Control arm:** Standard Colonoscopy Counselling<br><br>Randomized participants will receive standard counselling delivered by trained medical personnel for bowel preparation prior to colonoscopy
**Key Eligibility Criteria**	**Inclusion Criteria:**<br><br>1) All scheduled colonoscopy with indication<br><br>2) Adequate digital literacy<br><br>3) Adequate language literacy with Malay and English language<br><br>**Exclusion Criteria:**<br><br>1) Patients with memory impairment due to previous stroke, dementia or Alzheimer's disease<br><br>2) Diagnosed with clinical anxiety
**Trial Type**	Purpose: Diagnostic<br><br>Allocation: Randomized Controlled Trial<br><br>Framework: Non-Inferiority<br><br>Masking: Single Masked [Outcome assessors (Endoscopists)]<br><br>Assignment: Parallel<br><br>Type of endpoint: Efficacy<br><br>Phase: NA (Diagnostic Trial)

**Date of First Enrolment**	01/04/2025
**Sample Size**	43 participants per arm (n_{total} = 86 participants)<br><br>Including 10% drop-out: 48 participants per arm (n_{total} = 96 participants)
**Recruitment Status**	Recruiting
**Primary Outcome**	**Bowel Preparation Quality:** Measured by the Boston Bowel Preparation Score (BBPS) [range for total scores: 0 (poor) to 9 (excellent)) taken from three colonic segments (right, transverse and left colon; each segment will be scored using BBPS scores of between 0 (poor) to 3 excellent] during colonoscopy.
**Secondary Outcomes**	1) **Patient Anxiety Score:** Measured by the English and Malay-translated and validated Depression Anxiety and Stress Scale-21 (DASS-21). Anxiety level will be assessed on the day of the procedure (pre-colonoscopy), reflecting state anxiety after the intervention versus baseline expectations.<br><br>2) **Patient Satisfaction:** Measured by the English and Malay-translated and validated Patient Satisfaction Questionnaire-18 (PSQ-18) immediately after the intervention (on the day of colonoscopy). This captures satisfaction with the preparation process and information received.<br><br>3) **Adequate Bowel Preparation Rate:** Proportion of patients achieving adequate BBPS (score ≥2 in all three colonic segments)<br><br>4) **Other Colonoscopy Quality Metrics:** Including polyp detection rate, adenoma detection rate and cecal intubation rate, to contextualize preparation quality.
**Ethics Review**	**Status:** Approved<br><br>**Approval Date:** 14-03-2025

	**Approval ID:** UKM PPI/111/18,<br><br>**Contact:** UKM Research Ethics Committee
**Data Sharing Statement**	Anonymized and de-identified participants' clinical and trial outcome data will be shared through the Harvard Dataverse Repository (https://dataverse.harvard.edu/). The full statistical codes used for trial data cleaning, transformation and analysis will be made available on the repository.

N/A: Not Available; WHO: World Health Organization

Table 3. Relevant participant demographics will be collected during the trial.

**Items**	**Descriptions**
**Section 1: Patient Demographics**	
Name	
Age (in years)	
Gender	
Body Mass Index (BMI, in kg/m^2)	
Profession / Occupation	

Digital Literacy Level	Basic/Intermediate/Advanced
Comorbidities	Diabetes Mellitus/Hypertension/Chronic Kidney Disease/Ischemic Heart Disease<br><br>Others:
Relevant medical history	Previous history of constipation (Rome's Criteria)/ Previous history of diarrhoea<br><br>Others:
Smoker	Yes/No
Alcohol drinker	Yes/No
**Section 2: Bowel Preparation**	
Dietary restriction before colonoscopy	Yes/No
Completion of laxative (Fortrans^{TM}) as instructed	Yes/No<br><br>If No state the reasons:

**Section 3: Colonoscopy Details**	
Indication for colonoscopy	
Dosage of sedation (IV Midazolam in mg & IV Pethidine in mg)	
Completion of colonoscopy	Complete/Incomplete<br><br>If Incomplete, state the reason:
Time taken (in minutes)	
Withdrawal time (in minutes)	
Diagnosis	
Bowel preparation method	
Boston Bowel Preparation Scores (BBPS)	
Finding(s)	
Intervention during colonoscopy	(Yes/No)

	If yes state, the indication:<br>• Biopsy<br>• Polypectomy<br>• Others: ___________________
Histopathology examination (if available).	Benign/Malignant.
Adverse event	Yes/No<br><br>If yes, state the event:
Baseline Anxiety Level (DASS-21 score)	

Study Setting

The trial will be conducted at Hospital Canselor Tuanku Muhriz (HCTM), the teaching hospital of The National University of Malaysia (UKM) in Cheras, Kuala Lumpur. Patient recruitment will be carried out between 01/04/2025 and 31/07/2025.

Eligibility Criteria

Individuals who wish to take part in this study must meet several inclusion criteria. They need to be at least 18 years old and scheduled for a first-time or repeat elective colonoscopy at Hospital Canselor Tuanku Muhriz (HCTM). Besides, participants should be comfortable using a

smartphone, tablet, or computer to interact with a web-based system, have basic digital literacy and have personal internet access (provided by HCTM if needed). They also need to be able to understand and communicate in either English or Bahasa Malaysia, as the chatbot used in the study will rely on these languages. Lastly, they must be prescribed a standard bowel preparation regimen by their attending doctor, without any special adjustments.

At the same time, the following individuals will be excluded from the trials: People with significant cognitive problems—such as dementia or serious neurological conditions—that affect their ability to follow instructions, those who require a customized bowel preparation, for example due to chronic kidney disease, inflammatory bowel disease, or similar conditions, those with severe anxiety disorders or other psychiatric illnesses that could affect how they experience or report anxiety and individuals who are not able—or do not consent to study participation.

Randomization and Masking

After eligibility screening and informed consent, participants will be randomly allocated to the AI chatbot arm or control arm. A computer-generated random sequence (blocks of 4) will be created by the trial statistician (MIAJ) for assigning participants to the allotted interventions in a 1:1 ratio and this will be stored in the REDCap system. All other study personnel will not have access to the randomization sequence to maintain allocation concealment. Recruitment will be mainly performed by the principal investigator (NMA). The REDCap system will only be used by the study personnel to allocate participants to trial interventions after participants have consented to trial participation. The trial statistician (MIAJ) will neither be involved in participant recruitment nor in the administration of the allotted intervention.

The endoscopist performing the colonoscopy and scoring the BBPS should have 5 years of experience and have carried out at least 200 colonoscopies per year. Participants and other study personnel will not be masked to the assigned interventions, but outcomes (BBPS, anxiety, satisfaction) will be objectively assessed by endoscopists who will be masked to the interventions received by the participants. The endoscopists and participants will be instructed to maintain minimal conversation to prevent unmasking. No emergency unmasking is anticipated since we do not expect any significant harms to be associated with the interventions received by the participants.

Interventions

Control Arm (Standard Counseling)

Participants in the control group will receive routine pre-procedure counseling as per hospital protocol. This includes written instructions (as a brochure) and/or face-to-face explanation by trained healthcare staff about dietary restrictions and bowel preparation steps. The counseling covers key points on diet modification and laxative use (Polyethylene glycol 4000 (Fortrans™);

DCH-Auriga, Malaysia) where 1 sachet of Fortrans^{TM} will be mixed with 1 liter of water and taken at 6:00 PM and 8:00 PM the night before, and another 1 L at 6:00 AM on the colonoscopy procedural day. Standard practice may include answering patient questions in person but does not involve any AI tools.

Intervention Arm (AI-ChatGPT Chatbot Counseling)

Participants in the intervention group will receive a supplementary counseling session via an AI chatbot powered by ChatGPT. The chatbot content is customized for colonoscopy preparation and is based on reputable guidelines from the European Society of Gastrointestinal Endoscopy (ESGE) and Malaysian Ministry of Health (MOH) recommendations. Each participant will first be given a personalized script (electronic or printed) that contains their name, age, relevant comorbidities, current medications, and scheduled procedure date. This script also summarizes the standard preparation protocol for clarity. Participants choose their preferred language (English or Malay), and the script is provided in that language.

After reviewing the script, participants will access the ChatGPT-based chatbot on a hospital-provided laptop (or personal device) in a private area. The chatbot is implemented via the OpenAI web interface (using ChatGPT version 4.5 "Orion", the latest available model). The chatbot's parameters are fixed (temperature = 1.0 for creative and empathetic responses, max tokens = 200,000) and each user's query will be submitted as a new session to prevent context carryover. A prompt engineering framework ensures consistency of responses.

The chatbot interaction proceeds as follows: the participant inputs their personalized script, and then the chatbot provides step-by-step guidance on dietary restrictions (e.g., clear fluid allowance, solid food cutoff) and bowel preparation (laxative timing and use). The chatbot also offers motivational encouragement and reinforcement messages. Participants may ask the chatbot additional questions about the procedure and preparation; the chatbot will answer within predefined safety limits (e.g., it can clarify instructions but will not give medical diagnoses). No PII is shared with ChatGPT; all interactions are captured via anonymized user inputs. If any user input is incomplete or unclear, the system's input validation will prompt for clarification. Clinic staff are available to assist with technology, but will not interject into the chatbot responses. Each chatbot session is expected to last 15–30 minutes.

The instructions for bowel preparation delivered by the ChatGPT chatbot will be developed through an iterative process: Clinical guidelines (ESGE, MOH) will be used to construct initial patient questions and corresponding answers. We will subsequently employ ChatGPT to draft dialogue scripts in both English and Malay. Multiple refinements ensured localization (e.g., replacing "pencahar" with "pelawas" in the Malay language, specifying local timing for laxative doses at 6 PM/8 PM and 6 AM/9 AM) and alignment with institutional practice. The final script will be structured into conversational sections (Introduction, Dietary Preparation, Bowel Preparation, Mental Preparation, Risks & Safety, Day of Procedure) to mirror typical patient

concerns. The content will be reviewed by clinicians for accuracy, cultural appropriateness, and readability before deployment. Thus, ChatGPT's knowledge base consists of standardized, evidence-based bowel preparation protocols, with patient-friendly language. The readability of the responses generated by ChatGPT will be assessed using the Flesh-Kincaid Grade Level score and the Gunning-Fog index [22, 23].

Those who cannot complete the structured input forms or who submit incomplete, non-English/non-Malay, or ambiguous responses will be identified during enrollment and will be referred to standard counselling instead (data from such cases will not enter AI analysis). The chatbot will not collect personal identifiable information (PII), and responses longer than 500 words (~800 –1000 tokens) will be truncated to fit processing constraints.

In this trial, ChatGPT is used only to support standard care by providing text-based bowel-preparation advice. Clinicians retain full responsibility for all decisions and will decide when in-person counselling is needed. If a patient raises questions that ChatGPT cannot answer, or if the information provided is not adequate for safe decision-making, the clinician in charge will step in and deliver conventional, in-person counseling.

AI System Infrastructure and Privacy

The chatbot is hosted on a secure cloud platform with strong data protection. Access is restricted to trial participants and authorized study personnel through authentication controls. All data transmissions are encrypted end-to-end. The ChatGPT system logs interaction data (timestamps, user inputs, and chatbot responses) for analysis, but no identifiable patient information is ever stored. Input restrictions (English/Malay text only, structured formats) ensure compatibility with the model. Responses exceeding the token limit are automatically truncated or rephrased by the system.

Clinical sites will have internet-enabled devices (computers, tablets, or smartphones) available in private rooms for supervised interactions if needed. Printed or digital instructions will orient participants on using the chatbot platform. Patients may also access the chatbot remotely via their own devices to review the information later. Technical support will be available to troubleshoot any access issues.

Algorithm Details and Updates

The AI chatbot uses the OpenAI's ChatGPT-4.5 model (Orion) (Pro Subscription). The deployed model version and system parameters (temperature, token limit) will be documented. If OpenAI releases updates during the trial period, we will record any change in model version and assess for consistency. Since ChatGPT is a proprietary model, its internal weights are not modifiable; we rely on configuring prompts and contexts. Our focus is on standardizing inputs and validating outputs to achieve reproducibility.

Human–ChatGPT Interaction and Expertise

The intervention is designed for direct patient use without real-time expert oversight. Participants need only basic digital literacy. Healthcare staff will introduce the chatbot and assist with any technical difficulties or initial login. They will not censor or alter the chatbot's responses but will be available to clarify instructions if the participant requests additional explanation. No specialized training in AI is required for staff or participants.

Error Monitoring and Performance Evaluation

Given the novel AI-ChatGPT intervention, we will systematically monitor chatbot performance. The study will classify any AI-generated errors (e.g., incorrect guidance, omissions, ambiguous responses) by frequency and type. Automated system logs will detect anomalies or repeated errors in output patterns. Additionally, at least 10% of chatbot sessions (randomly selected) will be reviewed by clinicians to assess the accuracy and appropriateness of the guidance. Participants can also flag any confusing or incorrect responses, which will be recorded. We will calculate error rates (e.g., percentage of responses flagged) and perform qualitative case reviews to identify common issues.

To mitigate safety risks, the chatbot's knowledge base is restricted to evidence-based bowel prep content. Potential hallucinations (fabricated or unsafe advice) are minimized by configuring the prompt context to clinical guidelines and by implementing real-time checks: if the chatbot's output falls outside established instructions or is contradictory, it will be logged and corrected offline. In practice, if a response appears clinically inappropriate (e.g., suggesting contraindicated actions), the participant will be directed to human counseling immediately. An escalation protocol ensures that any ambiguous or safety-related queries are referred to staff. These measures are intended to catch and correct AI errors before they impact patient care.

Bias, Explainability, and Reproducibility

We will actively address common AI concerns. The chatbot's training and prompts are based on standardized clinical protocols to reduce hallucinations or omissions. Apart from those, during the prompt engineering phase, we will also use the shortest possible prompts based on formal languages with maximum use of concrete words representing tangible objects to reduce ChatGPT's hallucination rate [24]. Besides, the ChatGPT system is tested to operate within defined knowledge boundaries (preventing speculative answers). We will conduct periodic audits of AI advice across demographic subgroups to check for any bias (e.g., differences in style or content by patient age or language). If any bias or disparity is found (for example, if the AI inadvertently gives different instructions for Malay vs. English outputs), we will adjust the model prompts accordingly.

The interaction framework is standardized: inputs are structured (same script template) and outputs follow fixed templates (sections on diet, instructions, etc.). This consistency enhances reliability and reproducibility. We will document the exact prompt templates, session settings, and output formats to enable replication. In addition, patient-facing language is kept clear and conversational to aid explainability. Participants can request simpler explanations from the chatbot if needed.

Data Protection and Privacy for ChatGPT-based intervention development

All AI interactions are designed with privacy safeguards. No PII is entered into the chatbot system. Data transmissions are encrypted, and the platform enforces access controls. Participant responses are de-identified before storage; we will use de-identification techniques to ensure that logged transcripts cannot be traced back to individuals. All data analysis will use anonymized IDs.

Reproducibility and Access to Intervention Details

ChatGPT 4.0 is a proprietary model not available for modification or distribution. Therefore, to support reproducibility, we will fully describe the intervention’s configuration: model version, system parameters, prompt designs, and supervision procedures. Study findings, including the scripts used and performance results, will be published. While the chatbot code cannot be shared, researchers with appropriate licenses could replicate the approach by following our documented methods. Anonymized interaction data (without PII) may be made available to other researchers upon reasonable request and institutional approval.

Trial endpoint assessments

The primary endpoint of this study is the quality of bowel preparation, assessed using the Boston Bowel Preparation Score (BBPS) during colonoscopy. This scoring system evaluates three segments of the colon—right, transverse, and left—with each segment scored from 0 to 3, resulting in a total score ranging from 0 (indicating poor preparation) to 9 (indicating excellent preparation). The evaluation will be carried out by endoscopists who are blinded to group allocation. Each patient’s total BBPS score will serve as the primary outcome measure for comparing bowel preparation quality across study arms.

Several secondary endpoints will also be assessed. Patient anxiety will be measured using the anxiety subscale of the Depression Anxiety and Stress Scale-21 (DASS-21), administered on the day of the colonoscopy to evaluate state anxiety following the intervention. Patient satisfaction will be assessed using the Patient Satisfaction Questionnaire-18 (PSQ-18), completed immediately after the procedure to capture perceptions of the preparation process and the clarity of information provided. The proportion of patients achieving adequate bowel preparation—defined as a BBPS score of at least 2 in all three segments—will be calculated to determine overall effectiveness. Additional exploratory outcomes will include standard colonoscopy quality metrics such as the

adenoma detection rate (ADR) and cecal intubation rate, providing context for interpreting the impact of preparation quality on procedural performance.

All questionnaires have been translated into Malay and have good properties of inter-rater reliability and validity: DASS-21 for anxiety (Cronbach's α: 0.81 (English) – 0.84 (Malay)) [26,27], and PSQ-18 for satisfaction (Cronbach's α: 0.745 (Malay) - 0.750 (English)) [28,29]. All secondary outcomes are scored on Likert scales for standardization. Anxiety and satisfaction are assessed at the time of the procedure when patient concerns are most salient, providing a realistic gauge of the intervention's impact.

Adverse event assessments and monitoring

Adverse events (AE) are defined as "an abnormal sign, symptom, laboratory test, syndromic combination of such abnormalities, untoward or unplanned occurrence (e.g., accident), or any unexpected deterioration of concurrent illness"[30]. For serious AE (SAE), this is defined as "adverse events that result in the following outcomes: 1) death; 2) life-threatening AEs; 3) inpatient hospitalization or prolongation of existing hospitalization; 4) a persistence of significant incapacity or substantial disruption [31].

Earlier research has demonstrated that the anticipated adverse events are essentially consistent with the complications typically associated with standard diagnostic and therapeutic colonoscopy procedures, such as abdominal bloating, flatulence, and the rarer but more serious adverse events, such as colonic perforation, infection, and post-polypectomy syndrome. Besides, the ChatGPT utilized in this study is an open-source large language model that does not involve direct contact with the human body. Therefore, ChatGPT is assumed to have minimal harm to study participants.

The participants will be monitored for 24 hours following colonoscopy for AE and SAE through clinical observations in the daycare ward and through telephone calls once the participants are discharged. All AEs and SAEs will be recorded in the case report form (CRF) and the details include the adverse event's characteristics, the date and time of onset and disappearance and severity of the AEs, which will be classified based on the Common Terminology Criteria for Adverse Events (CTCAE) Version 5 [32]. Participants experiencing SAEs will be treated via standard clinical management practice. All AEs grade 3 and above will be reported to the UKM Ethics Committee within five business days. On the other hand, SAEs (including Sudden Unexpected Serious Adverse Reactions (SUSAR)) will be reported to the UKM Ethics Committee within 24 hours (expedited reporting).

Sample Size Calculation

The sample size is based on detecting a difference in BBPS scores, which was calculated using PS Software version 3.1.2 (Dupont and Plummer, 2014, available from: https://github.com/vubiostat/ps/raw/refs/heads/master/bin/pssetup3.exe). We utilized the information from a similar trial by Zhu and colleagues [33]. The standard deviation of the BBPS in the standard instruction group was chosen to be 1.81, based on Zhu and co-workers [33]. We also assumed a minimum clinically important difference of BBPS between the ChatGPT-guided and standard instruction groups to be at least 1.1 BBPS points. Using a two-sample t-test (type I error rate (two-sided) = 0.05, power (1-β) = 80%) and allocation ratio (m) of 1:1, 43 patients per group (n_{total} = 86) are required. Considering a 10% attrition rate due to failure to obtain consents in eligible participants, participant withdrawal prior to randomization and others, the sample size is inflated to 48 participants per group (n_{total} = 96). Hence, we plan to screen 96 potential participants for eligibility before randomization.

No formal sample size calculation was performed for anxiety or satisfaction scores due to limited prior data. These will be analyzed as secondary outcomes.

Participant Recruitment Strategy and Recruitment Monitoring

We will recruit eligible participants who are patients scheduled for elective colonoscopy at the Endoscopic unit of HCTM, who will be identified from the weekly endoscopic list. The patient's eligibility will be verified by medically qualified personnel, who must document this in the patient's medical records upon taking consent for colonoscopy. As an additional strategy to ensure adequate patient recruitment, the medical officers at the HCTM surgical outpatient clinics will be notified to inform the research team members if potentially eligible participants are identified during the outpatient appointments.

The progress of recruitment will be monitored using this traffic light criteria:

Green: 24 or more participants recruited and randomized per month, with less than 10% attrition rate.

Yellow: 15 – 23 participants recruited and randomized per month, with less than 10% attrition rate. Strategies are found to increase the recruitment rate to 24 or more participants per month (green level).

Red: Less than 15 participants recruited and randomized per month, with less than 10% attrition rate. No strategies are found that can result in a sustainable increase in the recruitment rate to the green level.

The trial will be stopped if the recruitment rate falls under the red category for the trial progress criteria. The trial will be cautiously monitored for two months if the recruitment rate is in the yellow category and will be stopped prematurely if the trial recruitment is within the yellow

category for two consecutive months. The progress of participant recruitment will be monitored by the Trial Monitoring Group (TMG) consists of the principal investigator and trial statistician. The TMR's recommendation on the trial continuation status will be relayed and discussed with the funder monthly. The recruitment flow of the study participants is summarized in Figure 1.

FIGURE 1 File (Uploaded separately)

Figure 1: The planned schematic flow of the clinical trial

Data Collection and Management

Participants' demographic data (age, gender, BMI, occupation, digital literacy), medical history (comorbidities, medication use, prior GI history, smoking/alcohol), and preparation adherence will be collected by the study investigators. For intervention patients, chatbot interaction logs (questions asked and responses given) will be collected anonymously. Individual participant data (including outcome data) will be entered into a paper-based clinical record form (CRF; available upon request to the principal trial investigator (NMA)), which will be subsequently entered into a secure SPSS spreadsheet with coded personal identifiers. Only authorized study staff will have access to the data. All data manipulation will be performed using SPSS.

BBPS will be recorded by the endoscopist immediately after colonoscopy using the standard BBPS form. Anxiety and satisfaction questionnaires will be administered by the trained study investigators on the procedure day before sedation. The study investigators will be trained by an independent psychometrician via supervised interviews of potential respondents.

To preserve data quality control, data quality checks (range, consistency) will be performed regularly by the principal investigator and trial statistician. The research personnel collecting the data will also be instructed to evaluate their own performance by checking their individual rate of missing data and implausible responses. Any rectification of errors in data recording is only allowed before the information in the CRFs is transferred into the SPSS spreadsheet. The double data entry procedure will be carried out by two research personnel to prevent errors in data entry.

Besides, missing data for primary and secondary outcomes will be minimized by protocol diligence; all endoscopists will be consistently reminded to record the BBPS score and all colonoscopy quality metrics before performing colonoscopy. Besides, the participants will also be reminded by the trained questionnaire administrators to complete the whole questionnaire and all

completed questionnaire forms will be checked for response completion before colonoscopy is carried out.

Data Security and Storage

The personal identifying information for each participant will be removed from the SPSS spreadsheet. All CRFs will be stored in a designated locked cabinet accessible to the principal investigator and trial statistician only. All electronic datasets will be password protected and encrypted, and the encryption key will be kept by the trial statistician. All CRFs and SPSS datasets will be stored for at least 10 years after the end of the trial and routine data inspections will be made to ensure data readability.

Statistical Analysis Plan

Analysis will primarily follow the intention-to-treat (ITT) principle, where all randomized trial participants will be included in the analysis according to their original allocated intervention group. All anonymized and deidentified individual participant data and statistical codes used for the data cleaning, transformation and analysis will be shared via the Harvard Dataverse Repository (https://dataverse.harvard.edu/). Statistical analysis will be conducted using SPSS Version 29 (IBM Corp, Armonk, New York, USA; 2023) or R Version 4.5.1 (R Core Team, Vienna, Austria; 2025) software.

Baseline characteristics will be summarized by group means (standard deviation) or medians (interquartile range) for continuous variables and counts and percentages for categorical variables (e.g. adequate bowel preparation rate). The normality of the continuous variables will be assessed using objective (Shapiro-Wilks test, Fisher's coefficient of skewness [34]) and graphical measures (QQ Plot, Box and Whisker Plot).

Multiple imputations will be carried out if the percentage of missing data is more than 5% per variable under the missing at random (MAR) assumption using multiple imputation by chained equations method implemented on R "mice" package [35, 36]. Following recommended practice, 20 imputed datasets will be generated, and results will be pooled using Rubin's rules, which account for both within-imputation and between-imputation variability to obtain valid statistical inference and reduce Monte Carlo error [37, 38].

The primary analysis will univariably compare mean total BBPS between the AI-ChatGPT and standard counseling groups using an independent t-test (if normally distributed) or Mann-Whitney U test otherwise. Similarly, rates of adequate preparation (BBPS ≥6 overall or ≥2 per segment) will be compared with a chi-square test or Fisher's exact test if the number of cells in the contingency tables with expected counts of less than 5 is more than 20%. The non-inferiority margin (Δ) for BBPS score difference will be set at -1.1 points (i.e. the AI-ChatGPT arm is considered non-inferior if the lower bound of the two-sided 95% confidence interval (CI) for the

mean BBPS difference AI-ChatGPT – control is greater than -1.1 BBPS points) based on the minimum clinically important difference used for sample size calculation. For multivariable analysis stage, an analysis of covariance (ANCOVA) will be utilized to compare mean BBPS between AI-ChatGPT and standard counseling groups after adjusting for known confounders that influence bowel preparation adequacy such as age, gender (male as the risk factor), level of education, presence of diabetes mellitus, hypertension, history of constipation and opioid use [39, 40]. We will then evaluate the statistical assumptions of ANCOVA (residuals' normality and homoscedasticity, linearity of covariates with the outcome variables, homogeneity of regression slopes) and apply transformations if necessary.

For secondary outcomes, the adequate bowel preparation rate (BBPS ≥ 6 overall or ≥ 2 per colonic segment) will be compared using risk difference and two-sided 95% CI. The non-inferiority margin will be set at -10%. For change in anxiety (DASS-21) and satisfaction (PSQ-18) scores, ANCOVA will be employed with statistical adjustment made with baseline anxiety (DASS-21) and (PSQ-18) satisfaction scores. However, for these two secondary trial endpoints, the superiority of the AI-ChatGPT intervention over the standard counseling intervention will be tested. For colonoscopy quality metrics (ADR and cecal intubation rate), comparisons will only be made at the univariable level using the chi-squared or Fisher's exact test.

For sensitivity analysis, the results obtained based on the ITT analyses will be compared with the findings from the per-protocol (PP) analyses. Besides, if there are trial variables with a more than 5% rate of missingness, the results based on the imputed datasets will be compared with the findings from complete-case analyses. Apart from that, worst-case imputation for missing primary endpoint (control group had the best BBPS scores, AI-ChatGPT group had the worst) will also be employed to assess the robustness of the results. The conclusion will be based on concordant results (i.e., non-inferiority is observed in all sensitivity analyses) as recommended by the International Council for Harmonization (ICH) E9(R1) statistical principles [41].

For non-inferiority results, they will be reported as point estimates with corresponding 95% CIs and visually presented using the forest plots, with the non-inferiority margins (e.g., -1.1 BBPS points and -10% risk difference) will be clearly indicated as vertical reference lines. Two-sided p-value of 0.05 (for secondary/superiority tests) and one-sided p-value of 0.025 (for primary non-inferiority test) will be employed as the statistical significance thresholds.

Ethical Considerations and Safety

Participation is voluntary and all patients can withdraw at any time without affecting their care. The chatbot provides non-judgmental guidance, and no medications or invasive procedures are involved in the intervention itself. Should a participant exhibit significant distress or report an urgent medical concern during the chatbot session, staff will intervene according to clinical judgment. Any adverse events (e.g., severe anxiety spikes) related to the study procedures will be

recorded and reported to the ethics committee. Standard clinical practice will continue for all patients (for example, rescheduling a colonoscopy if bowel preparation is insufficient).

Protocol Amendments

Any modifications to this protocol (e.g., changes in chatbot implementation) will be submitted to the ethics committee for approval. The trial registry will be updated with protocol versions. Study progress (enrollment dates, recruitment numbers, withdrawals, and any serious events) will be communicated to the funder and oversight bodies per institutional requirements.

Dissemination of trial findings

Results will be published in peer-reviewed journals and presented at scientific meetings. The full protocol and statistical analysis plan will be made publicly available (e.g., ClinicalTrials.gov or institutional repository).

Trial Oversight and Monitoring

The trial steering committee comprises the trial principal investigators (NMA and MIAJ). It is responsible for the design, execution, and overall monitoring of the progress of the trial. Besides, the trial steering committee is also responsible for executing any trial modifications, including the ones proposed by the Data Monitoring Committee (DMC).

On the other hand, the trial management group (TMG) is responsible for ensuring a smooth conduct of the day-to-day trial operations. This comprises NMA (principal investigator) and other co-investigators (HA, IN, YJ, NG, JA and YSQ).

The DMC is appointed to monitor the trial safety data and comprises one independent statistician and a colorectal surgeon. All DMC members declared no conflict of interest.

Discussion

Effective bowel preparation is key to a high-quality colonoscopy. By leveraging AI, we aim to enhance patient education in a scalable way. The primary outcome, the BBPS, is a validated measure of bowel cleanliness [18], with higher scores reflecting clearer mucosa. Even modest improvements in BBPS can increase adenoma detection and reduce repeat procedures. Secondary outcomes (anxiety and satisfaction) address patient-centered effects: reduced anxiety and higher satisfaction may improve overall preparation adherence and experience.

This trial incorporates rigorous methods to ensure reliability and safety. Blinding of endoscopists and validated scales (BBPS, DASS-21, PSQ-18) promote unbiased outcome assessment. The sample size is powered for the expected effect on BBPS, and analyses will adjust for potential confounders.

Unique to this study is the detailed compliance with SPIRIT-AI guidelines. We have explicitly described the AI model version, input requirements, handling of bad input, human–AI interaction, and performance monitoring. We will analyze any AI performance errors and implement an escalation pathway if needed. Strategies to prevent hallucinations and bias include restricting content to evidence-based guidelines and auditing outputs. Privacy protections (no PII, encryption, access controls) safeguard patient data.

If successful, this study will demonstrate that an AI chatbot can be a viable adjunct to traditional counseling, improving colonoscopy preparation quality. It will also provide a model for the transparent reporting of AI interventions in clinical trials, per SPIRIT-AI. Further research will be needed to generalize findings to other settings, but this trial aims to set a new standard for integrating AI safely into patient education.

Budget Proposal

Equipment and Supplies

No.	Item	Quantity	Unit Cost (RM)	Total Cost (RM)	Justification
1.	Chat Gpt Plus	6 month	$25 (RM111.75)/ month	RM 671	ChatGPT Plus ensures reliable access, minimizing disruptions and response variability. It restricts sources to trusted, Malaysia-approved resources for accurate colonoscopy guidance. With enhanced reliability and real-time support, it maintains study integrity, ensures high-quality research, and prioritizes patient safety with medically approved advice from authoritative sources.
2.	Patient Honorarium	96 patient	RM 10/patient	RM 960	The patient honorarium budget values participants' time, encouraging enrollment and adherence. It boosts engagement, reduces dropouts, and enhances data reliability, ensuring the study's successful completion and valid findings
	Total			RM 1631	

*Based on current currency

Source: Geran Fundamental Fakulti Perubatan

Gantt Chart

No.	Task/Month	Oct	Nov	Dec	Jan	Feb	March	Apr	May	June	July	Aug	Sept
1.	First meeting with supervisor and discuss possible title for research	■											
2.	Discuss literature review related with research title		■										
3.	Budget proposal discussion		■										
4.	Submission of proposal			■									
5.	Present proposal at the department				■								
6.	Submit proposal to ethics committee				■								
7.	Present the proposal to the ethics committee if needed.				■								
8.	Data collection					■	■	■	■	■			
9.	Prepare manuscript and Data Analysis										■	■	
10.	Manuscript submission											■	

Appendix 1

Proforma Form

**Section 1: Patient Demographics**	
Name	
Age	
Gender	
MRN	
BMI	
Profession / Occupation	
Digital Literacy Level	Basic/Intermediate/Advanced
Comorbidities	Diabetes Mellitus/Hypertension/Chronic Kidney Disease/Ischemic Heart Disease<br><br>Others:
Relevant medical history	Previous history of constipation (Rome’s Criteria)/ Previous history of diarrhoea<br><br>Others:
Smoker	Yes/No
Alcohol drinker	Yes/No
**Section 2: Bowel Preparation**	
Dietary restriction before colonoscopy	Yes/No
Completion of laxative (Fourtrans) as instructed	Yes/No<br><br>If No state the reasons:
**Section 3: Colonoscopy Details**	
Indication for colonoscopy	

Dosage of sedation(midazolam&pethidine)	
Completion	Complete/Incomplete<br><br>If Incomplete state the reason:
Time taken	
Diagnosis	
Bowel preparation method	
Boston Bowel Preparation Scores (BBPS)	
Finding	
Intervention during colonoscopy	(Yes/No)<br>If Yes state the indication:
Histopathology examination (if available).	Benign/Malignant.
Adverse event	Yes/No<br><br>If Yes state the event:

Section 4: Surveys and Questionnaires

- DASS 21
- Patient Satisfaction Questionnaire -18

Appendix 2

Personalized scripts

<u>English</u>

Hi, my name is **[Insert Name]**. I am **[Insert Age]** years old and have the following medical conditions: **[List Comorbidities Ex: Diabetes, Cholesterol, Blood Pressure]**. I am currently on these medications: **[List Medications]**. I am scheduled for a colonoscopy on **[Insert Date]**. Please ensure that all the answers related with the colonoscopy procedure will be from KKM guidelines and a reputable journal. I am using fortrans for laxative.

Can you tell me what I need to do or know before the colonoscopy?

<u>Malay</u>

Hai, nama saya **[Masukkan Nama]**. Saya berumur **[Masukkan Umur]** tahun dan mempunyai keadaan kesihatan seperti berikut: **[Senaraikan Komorbiditi, CTH : Kencing manis, Kolesterol, Darah Tinggi].** Saya sedang mengambil ubat-ubatan ini: **[Senaraikan Ubat]**. Saya dijadualkan untuk kolonoskopi pada **[Masukkan Tarikh].** Sila pastikan bahawa semua jawapan berkaitan prosedur kolonoskopi adalah berdasarkan garis panduan KKM (Kementerian Kesihatan Malaysia) dan jurnal yang bereputasi. Saya menggunakan Fortrans un

Boleh tak anda terangkan apa yang saya perlu buat atau tahu sebelum kolonoskopi?

References

1. Atkin W, Wooldrage K, Parkin DM, Kralj-Hans I, MacRae E, Shah U, Duffy S, Cross AJ. Long term effects of once-only flexible sigmoidoscopy screening after 17 years of follow-up: the UK Flexible Sigmoidoscopy Screening randomised controlled trial. Lancet. 2017 Apr 1;389(10076):1299-1311. doi: 10.1016/S0140-6736(17)30396-3.

2. Hassan C, East J, Radaelli F, Spada C, Benamouzig R, Bisschops R, Bretthauer M, Dekker E, Dinis-Ribeiro M, Ferlitsch M, Fuccio L, Awadie H, Gralnek I, Jover R, Kaminski MF, Pellisé M, Triantafyllou K, Vanella G, Mangas-Sanjuan C, Frazzoni L, Van Hooft JE, Dumonceau JM. Bowel preparation for colonoscopy: European Society of Gastrointestinal Endoscopy (ESGE) Guideline - Update 2019. Endoscopy. 2019 Aug;51(8):775-794. doi: 10.1055/a-0959-0505.

3. Alvarez-Gonzalez MA, Flores-Le Roux JA, Seoane A, Pedro-Botet J, Carot L, Fernandez-Clotet A, Raga A, Pantaleon MA, Barranco L, Bory F, Lorenzo-Zuñiga V. Efficacy of a multifactorial strategy for bowel preparation in diabetic patients undergoing colonoscopy: a randomized trial. Endoscopy. 2016;48(11):1003-1009. doi: 10.1055/s-0042-111320.

4. Mahmood S, Farooqui SM, Madhoun MF. Predictors of inadequate bowel preparation for colonoscopy: a systematic review and meta-analysis. Eur J Gastroenterol Hepatol. 2018;30(8):819-826. doi: 10.1097/MEG.0000000000001175.

5. Zhou J, Wu L, Wan X, Shen L, Liu J, Zhang J, Jiang X, Wang Z, Yu S, Kang J, Li M, Hu S, Hu X, Gong D, Chen D, Yao L, Zhu Y, Yu H. A novel artificial intelligence system for the assessment of bowel preparation (with video). Gastrointest Endosc. 2020 Feb;91(2):428-435.e2. doi: 10.1016/j.gie.2019.11.026.

6. Lim DYZ, Tan YB, Koh JTE, Tung JYM, Sng GGR, Tan DMY, Tan CK. ChatGPT on guidelines: Providing contextual knowledge to GPT allows it to provide advice on appropriate colonoscopy intervals. J Gastroenterol Hepatol. 2024;39(1):81-106. doi: 10.1111/jgh.16375.

7. Bibault JE, Chaix B, Nectoux P, Pienkowsky A, Guillemasse A, Brouard B. Healthcare ex Machina: Are conversational agents ready for prime time in oncology? Clin Transl Radiat Oncol. 2019;16:55-59. doi: 10.1016/j.ctro.2019.04.002.

8. Laranjo L, Dunn AG, Tong HL, Kocaballi AB, Chen J, Bashir R, Surian D, Gallego B, Magrabi F, Lau AYS, Coiera E. Conversational agents in healthcare: a systematic review. J Am Med Inform Assoc. 2018;25(9):1248-1258. doi: 10.1093/jamia/ocy072.

9. Luxton DD. Ethical implications of conversational agents in global public health. Bull World Health Organ. 2020 Apr 1;98(4):285-287. doi: 10.2471/BLT.19.237636.

10. Bibault J, Chaix B, Nectoux P, Pienkowsky A, Guillemasse A, Brouard B. Healthcare ex Machina: are conversational agents ready for prime time in oncology? Clin Transl Radiat Oncol 2019 May;16:55-59 doi: 10.1016/j.ctro.2019.04.002.

11. Davenport T, Kalakota R. The potential for artificial intelligence in healthcare. Future Healthc J 2019;6(2):94-98 doi: 10.7861/futurehosp.6-2-94.

12. Chang P, Sheng Y, Sang Y, Wang D. Developing a wireless speech- and touch-based intelligent comprehensive triage support system. Comput Inform Nurs 2008;26(1):31-38. doi: 10.1097/01.NCN.0000304754.49116.b4.

13. van Heerden A, Ntinga X, Vilakazi K. The Potential of Conversational Agents to Provide a Rapid HIV Counseling and Testing Services. In: International Conference on the Frontiers and Advances in Data Science. 2017 Presented at: FADS'17; October 23-25, 2017; Xi'an, China. doi: 10.1109/fads.2017.8253198

14. Bickmore TW, Pfeifer LM, Byron D, Forsythe S, Henault LE, Jack BW, et al. Usability of conversational agents by patients with inadequate health literacy: evidence from two clinical trials. J Health Commun 2010;15(Suppl 2):197-210. doi: 10.1080/10810730.2010.499991

15. Luxton DD. Ethical implications of conversational agents in global public health. Bull World Health Organ 2020;98(4):285-287 doi: 10.2471/BLT.19.237636

16. Zhang Z, Bickmore T. Medical Shared Decision Making with a Virtual Agent. In: Proceedings of the 18th International Conference on Intelligent Virtual Agents. 2018 Presented at: IVA'18; November 5-8, 2018; Sydney, NSW, Australia.

17. Milne-Ives M, de Cock C, Lim E, Shehadeh M, de Pennington N, Mole G, Normando E, Meinert E The Effectiveness of Artificial Intelligence Conversational Agents in Health Care: Systematic Review J Med Internet Res 2020;22(10):e20346, doi: 10.2196/20346.

18. Fitzpatrick KK, Darcy A, Vierhile M. Delivering Cognitive Behavior Therapy to Young Adults With Symptoms of Depression and Anxiety Using a Fully Automated Conversational Agent (Woebot): A Randomized Controlled Trial. JMIR Ment Health. 2017;4(2):e19. doi: 10.2196/mental.7785.

19. Jiang Y, Fu X, Wang J, Liu Q, Wang X, Liu P, Fu R, Shi J, Wu Y. Enhancing medical education with chatbots: a randomized controlled trial on standardized patients for colorectal cancer. BMC Med Educ. 2024;24(1):1511. doi: 10.1186/s12909-024-06530-8.

20. Hróbjartsson A, Boutron I, Hopewell S, Moher D, Schulz KF, Collins GS, et al. SPIRIT 2025 explanation and elaboration: updated guideline for protocols of randomised trials. BMJ. 2025;389:e081660. doi: 10.1136/bmj-2024-081660.

21. Rivera SC, Liu X, Chan AW, Denniston AK, Calvert MJ; SPIRIT-AI and CONSORT-AI Working Group. Guidelines for clinical trial protocols for interventions involving artificial intelligence: the SPIRIT-AI Extension. BMJ. 2020;370:m3210. doi: 10.1136/bmj.m3210.

22. Kincaid JP, Fishburne RP, Rogers RL, Chissom BS. Derivation of new readability formulas (Automated Readability Index, Fog Count and Flesch Reading Ease Formula) for Navy enlisted personnel. Research Branch Report 8-75. Millington (TN): Naval Air Station Memphis, Chief of Naval Technical Training; 1975.

23. Gunning R. The technique of clear writing. New York: McGraw-Hill; 1952.

24. Rawte V, Priya P, Tonmoy SMTI, Zaman SMMM, Sheth A, Das A. Exploring the Relationship between LLM Hallucinations and Prompt Linguistic Nuances: Readability, Formality, and Concreteness. Computer Science: Artificial Intelligence. 2023. doi: 10.48550/arXiv.2309.11064.

25. Lai EJ, Calderwood AH, Doros G, Fix OK, Jacobson BC. The Boston bowel preparation scale: a valid and reliable instrument for colonoscopy-oriented research. Gastrointest Endosc. 2009;69(3 Pt 2):620-5. doi: 10.1016/j.gie.2008.05.057.

26. Lovibond PF, Lovibond SH. The structure of negative emotional states: comparison of the Depression Anxiety Stress Scales (DASS) with the Beck Depression and Anxiety Inventories. Behav Res Ther. 1995;33(3):335-43. doi: 10.1016/0005-7967(94)00075-u.

27. Musa R, Fadzil MA, Zain Z. Translation, Validation and Psychometric Properties of Bahasa Malaysia Version of the Depression Anxiety and Stress Scales (DASS). ASEAN Journal of Psychiatry. 2007;8(2):82-89.

28. Marshall GN, Hays RD. The Patient Satisfaction Questionnaire Short Form (PSQ-18). Santa Monica, CA: RAND Corporation; 1994. p. 7865.

29. Chan CM, Azman WA. Attitudes and role orientations on doctor-patient fit and patient satisfaction in cancer care. Singapore Med J. 2012;53(1):52-6.

30. Aronson JK. Medication errors: what they are, how they happen, and how to avoid them. QJM. 2009;102(8):513-21. doi: 10.1093/qjmed/hcp052.

31. Code of Federal Regulation, Title 21, Section 312.32 (21CFR312.32). (2020). Accessible from: https://www.accessdata.fda.gov/scripts/cdrh/cfdocs/cfcfr/cfrsearch. cfm?fr=312.32. Date of access: 27th May 2025.

32. U.S. Department of Health and Human Services, National Institutes of Health, National Cancer Institute. Common Terminology Criteria for Adverse Events (CTCAE) Version 5.0. Bethesda, MD: National Cancer Institute; 2017 Nov. Available from:https://ctep.cancer.gov/protocoldevelopment/electronic_applications/docs/CTCAE_v5_Quick_Reference_8.5x11.pdf. Date of access: 27th May 2025.

33. Zhu Y, Zhang DF, Wu HL, Fu PY, Feng L, Zhuang K, et al. Improving bowel preparation for colonoscopy with a smartphone application driven by artificial intelligence. NPJ Digit Med. 2023;6(1):41. doi: 10.1038/s41746-023-00786-y.

34. Doane DP, Seward LE. Measuring skewness: a forgotten statistic? J Stat Educ. 2011;19(2):1–18.

35. Jakobsen JC, Gluud C, Wetterslev J, Winkel P. When and how should multiple imputation be used for handling missing data in randomised clinical trials - a practical guide with flowcharts. BMC Med Res Methodol. 2017;17(1):162. doi: 10.1186/s12874-017-0442-1.

36. van Buuren S, Groothuis-Oudshoorn K. mice: Multivariate imputation by chained equations in R. J Stat Softw. 2011;45(3):1–67. doi:10.18637/jss.v045.i03.

37. Rubin DB. Multiple Imputation for Nonresponse in Surveys. 3rd ed. Hoboken (NJ): John Wiley & Sons; 2022.

38. White IR, Royston P, Wood AM. Multiple imputation using chained equations: issues and guidance for practice. Stat Med. 2011;30(4):377–99. doi:10.1002/sim.4067.

39. Mahmood S, Farooqui SM, Madhoun MF. Predictors of inadequate bowel preparation for colonoscopy: a systematic review and meta-analysis. Eur J Gastroenterol Hepatol. 2018;30(8):819-826. doi: 10.1097/MEG.0000000000001175.

40. Chan WK, Saravanan A, Manikam J, Goh KL, Mahadeva S. Appointment waiting times and education level influence the quality of bowel preparation in adult patients undergoing colonoscopy. BMC Gastroenterol. 2011;11:86. doi: 10.1186/1471-230X-11-86.

41. International Council for Harmonisation. Addendum on estimands and sensitivity analysis in clinical trials to the guideline on statistical principles for clinical trials E9(R1). ICH Harmonised Guideline. ICH E9(R1). Geneva: ICH; 2019.
