# Supplementary material for: A prospective single-masked, non-inferiority, parallel-group randomized controlled trial of the efficacy of a ChatGPT-based AI chatbot to improve Boston bowel preparation scores for colonoscopy preparation: A trial protocol
Source: PLoS One. 2025 Oct 15;20(10):e0334349. doi: 10.1371/journal.pone.0334349 (PMC12527164; doi:10.1371/journal.pone.0334349)
Supplement: Attachment — Submitted filename: Response to Reviewers (PONE-D-25-33008).docx [file pone.0334349.s004.docx]

Date: 10^th^ September 2025

Editorial Board,

PLOS One

Dear Editor,

We are profoundly grateful to you and the reviewers for the constructive and insightful feedback on our manuscript. I am herewith pleased to resubmit our revised manuscript entitled **“A Prospective Single-Masked, Non-Inferiority, Parallel-Group Randomized Controlled Trial of the Efficacy of a ChatGPT-Based AI Chatbot to Improve Boston Bowel Preparation Scores for Colonoscopy Preparation: A Trial Protocol”** (**Manuscript ID: PONE-D-25-33008**) for further review. We have carefully addressed every comment point-by-point (**Appendix A**), incorporating clarifications, protocol revisions, and additional literature support where appropriate. All amendments have been made using **American English** and formatted according to **Vancouver referencing style**. For clarity, the references cited in the rebuttal **retain the numbering from the main manuscript** **and follow the order of first citation in the main manuscript text.**

In cases where the reviewers raised methodological concerns based on incomplete assumptions, we have provided evidence-backed rebuttals while maintaining scientific rigor. No new data have been collected since the initial submission, and the study protocol remains conceptually consistent with our original design.

Below, we present our detailed responses to each reviewer’s comments. For each point, we:

1. Quote the reviewer’s comment.
2. Provide our detailed response.
3. Indicate the exact manuscript change (with section and line reference).
4. Include relevant references in Vancouver style.

We believe these revisions fully address the reviewers’ concerns and strengthen the protocol. Finally, this study is financially sponsored by the **Faculty of Medicine Fundamental Grant, UKM (Grant number: FF-2025-112)**. The **funders had no role in study design, data collection and analysis, decision to publish, or preparation of the manuscript**.

Kind regards,

**Muhammad Irfan Abdul Jalal [Corresponding Author]**

On behalf of all co-authors

Email: [irfan.abduljalal@ukm.edu.my](mailto:irfan.abduljalal@ukm.edu.my)

**"Towards Precision Medicine"**

**Appendix A**

**A) Reviewer 1: Detailed Point-by-Point Responses**

**1) Reviewer’s Major Comment 1:** **Clinical justification for -1.1 BBPS non-inferiority margin**

While the manuscript clearly defines the statistical rule for non-inferiority (i.e., the lower bound of the 95% CI for the BBPS difference must be greater than -1.1), the choice of the -1.1 BBPS point margin requires more clinical justification. The authors should elaborate why this value is considered clinically acceptable, e.g., by referencing the minimal clinically important difference (MCID), BBPS system validation, or its expected impact on detecting adenomas or determining adequacy (BBPS ≥6).

**i) Author’s Response:**

We appreciate the reviewer’s observation and agree that the justification for the non-inferiority margin should be grounded in robust clinical evidence. The -1.1-point non-inferiority margin for the Boston Bowel Preparation Score (BBPS) was selected based on published literature assessing the minimal clinically important difference (MCID) for the Boston Bowel Preparation Scale (BBPS) and its correlation with adenoma detection rate (ADR). Lai et al. validated the BBPS and found that adequate preparation, defined as a total BBPS score ≥6 with no segment <2, is associated with acceptable ADRs and caecal intubation rates [39]. Calderwood et al. demonstrated that differences of ≤1 point across BBPS total scores are not associated with clinically meaningful differences in ADR or polyp detection [40].

Furthermore, a non-inferiority margin of -1.1 BBPS points preserves the probability of maintaining an adequate bowel preparation rate above the 85% benchmark recommended by the European Society of Gastrointestinal Endoscopy (ESGE), since even after allowing for this margin the expected mean BBPS remains above the adequacy threshold (≥6) [25]. For example, if the control group mean is 7.5, applying the -1.1 BBPS margin still yields 6.4, which corresponds to at least 85% of patients achieving adequate bowel preparation. Setting a stricter margin would risk underpowering the study unnecessarily, whereas a wider margin could compromise clinical relevance. Our choice is therefore both statistically coherent and clinically conservative and meaningful.

**ii) Exact manuscript change:** **In Methods (Subsection Statistical Analysis)**

“The non-inferiority margin of -1.1 BBPS points was selected based on validation studies showing that differences of this magnitude do not compromise adenoma detection rate or adequacy classification (BBPS ≥6 with all segments ≥2) [39, 40]. This threshold is also consistent with ESGE guideline quality benchmarks that require at least 85% adequacy in bowel preparation [23].” (**pages 31-32; lines 595 - 600**)

**iii) References (citation order and number are as in the main manuscript):**

23. Hassan C, East J, Radaelli F, Spada C, Benamouzig R, Bisschops R, et al. Bowel preparation for colonoscopy: ESGE Guideline - Update 2019. Endoscopy. 2019;51(8):775–794. doi:10.1055/a-0959-0505.

39. Lai EJ, Calderwood AH, Doros G, Fix OK, Jacobson BC. The Boston bowel preparation scale: a valid and reliable instrument for colonoscopy-oriented research. Gastrointest Endosc. 2009;69(3 Pt 2):620-5. doi: 10.1016/j.gie.2008.05.057.

40. Calderwood AH, Schroy PC 3rd, Lieberman DA, Logan JR, Zurfluh M, Jacobson BC. Boston Bowel Preparation Scale scores provide a standardized definition of adequate for describing bowel cleanliness. Gastrointest Endosc. 2014;80(2):269–276. doi: 10.1016/j.gie.2014.01.031.

___________________________________________________________________________

**2) Reviewer’s major Comment 2: Risk of performance/detection bias with single masking**

The study is single-masked (endoscopist only), but the participants and staff interacting with them are unblinded. This introduces a risk of performance and detection bias, particularly in patient-reported outcomes (anxiety, satisfaction). Please discuss how this potential bias will be mitigated (for instance, standardizing questionnaire delivery).

**i) Author’s Response:**

We thank the reviewer for raising this important point. We acknowledge that in a single-masked trial, both participants and staff interacting with them are unblinded, which may raise concerns about performance and detection bias in patient-reported outcomes (anxiety, satisfaction). To mitigate this, we have implemented multiple safeguards, consistent with SPIRIT, CONSORT, and Cochrane guidance:

1. **Standardized administration** – All patient-reported outcomes (DASS-21 and PSQ-18) will be delivered by research assistants using a verbatim scripted protocol, independent of allocation.
2. **Electronic self-completion** – Patients will enter responses directly into a secure electronic system (REDCap®), eliminating interviewer influence and transcription errors.
3. **Training and monitoring** – Research assistants will undergo structured training, competency assessment, and ongoing monitoring. The trial coordinator will conduct random audits to ensure fidelity.
4. **Temporal and environmental standardization** – Questionnaires will be administered at fixed, prespecified time points (immediately pre- and post-colonoscopy) in a standardized quiet environment.
5. **Validated tools** – Both DASS-21 and PSQ-18 are validated in Malay and English, with high reliability, reducing susceptibility to contextual variation.

Regarding **patient blinding**, this is not feasible in our setting. The intervention being compared is AI chatbot counselling versus standard human counselling, and participants are necessarily aware of which modality they are receiving. Concealing this would be impractical, as patients can readily recognize whether they are interacting with an AI chatbot or a clinician. Thus, any attempt to conceal this would be artificial and unworkable.

Regarding **staff blinding**, only outcome assessors (endoscopists) are blinded, since they evaluate the primary endpoint (BBPS) during colonoscopy. Other staff members (e.g., study recruiters, research assistants administering questionnaires) cannot be blinded due to their direct role in facilitating either the chatbot session or the human counselling. However, their influence on outcomes is minimized by rigorous adherence to the trial protocol:

- Staff delivering counselling in the control arm follow a standardized hospital protocol.
- Research assistants administering questionnaires are not involved in providing counselling, and their interaction is limited to reading scripted instructions verbatim.
- Staff are trained to avoid non-verbal cues or discussion that could bias patient perceptions.

Therefore, while participants and some staff are necessarily unblinded, the measures above minimize the risk of systematic differences between trial arms. More importantly, the **primary endpoint (BBPS)** is objectively assessed by endoscopists who remain masked, ensuring the principal outcome of the trial is unbiased.

**ii) Exact manuscript changes:**

**a) In Methods (Subsections Trial Endpoint Assessments & Randomization and Masking)**

- “To minimize performance and detection bias, patient-reported outcomes (DASS-21, PSQ-18) will be administered by trained research assistants using a verbatim scripted protocol, without disclosure of allocation. Patients will complete questionnaires electronically, ensuring self-entry and eliminating interviewer influence. Research assistants will receive structured training and ongoing monitoring to ensure protocol fidelity. All questionnaires will be administered at prespecified time points in a quiet, standardized environment.” (**Page 22-23; Lines 422-428**)

- “Complete masking of participants and staff is not feasible since participants are inherently aware of whether they interact with an AI chatbot or a clinician, and staff facilitating the counseling or questionnaires necessarily know the intervention delivered. However, staff influence will be minimized through strict adherence to standardized hospital counseling protocols (control arm) and scripted questionnaire delivery (ChatGPT arms). Importantly, the BBPS is objectively evaluated by endoscopists who are masked to allocation, preserving the integrity of the primary endpoint evaluation. The endoscopists and participants will be instructed to maintain minimal conversation to prevent unmasking. No emergency unmasking is anticipated since we do not expect any significant harm to be associated with the interventions received by the participants.” (**Pages 14-15; Lines 179-189)**

**b) Discussion (Subsection Statistical Analysis)**

- “First, apart from the endoscopists who are masked, both participants and the staff facilitating counseling or questionnaire administration are necessarily unmasked. This design constraint arises because the nature of the intervention (AI chatbot vs. clinician counseling) is inherently perceptible to patients, and staff members must directly support the delivery of each counseling modality. However, the risk of bias from unblinded staff is minimized by strict adherence to the trial protocol: counseling in the control arm follows standard hospital protocols, and questionnaire administration in both arms is performed by trained research assistants using verbatim scripted instructions, with responses entered electronically by patients themselves. Most importantly, the primary outcome (BBPS) is assessed objectively by masked endoscopists, ensuring that the central efficacy measure remains unbiased.” **(Page 36; Lines 705-716)**

**___________________________________________________________________________**

**3) Reviewer’s Major Comment 3:** **The inclusion of only digitally literate patients and effects on selection bias and generalizability issues**

The inclusion of only digitally literate patients introduces a selection bias and limits generalisability, especially since poor bowel preparation is common among older adults, who may lack such literacy. This limitation should be more clearly acknowledged and discussed in the protocol.

**i) Author’s Response:**

We thank the reviewer for this important point. We acknowledge that restricting eligibility to participants with basic digital literacy and fluency in English or Malay may limit generalizability, particularly for older adults and those with reduced technology access, who are also populations at risk of inadequate bowel preparation.

This restriction was a deliberate and pragmatic choice for a first-in-context safety and feasibility trial. Early-phase evaluations of digital health and AI interventions commonly begin with populations capable of reliably engaging with the technology, in order to establish internal validity and safeguard content fidelity and safety before extending to broader populations [68-70].

To mitigate exclusion, participants without personal devices but otherwise eligible were provided with loaner tablets and a short onboarding session (≤10 minutes). Baseline digital literacy and language proficiency are recorded in the case report form, and these distributions will be reported descriptively in the results. We do not plan inferential subgroup analyses of digital literacy strata given the modest sample size, but transparent reporting will allow readers to judge representativeness. Future work will adapt the chatbot for low-literacy and non-English/Malay speakers through simplified interfaces, voice-based versions, and multilingual expansion.

**ii) Exact manuscript changes:**

**a) In Methods (Subsection Eligibility Criteria**)

“We require participants to have basic digital literacy in order to interact with the chatbot interface. For participants who are otherwise eligible but lack a personal device, the trial will provide a loaner tablet and a short onboarding session (10 minutes or less) to enable participation. The provision of loaner devices aims to mitigate but not eliminate selection bias; the trial remains a pragmatic first-phase evaluation among individuals who can use a mobile/tablet interface.” (**Page 13; Lines 149-155)**

**b) In Discussion (Subsection Study Limitations**)

Second, requiring basic digital literacy and fluency in English or Malay may limit generalizability of our study findings. This first-phase trial intentionally restricts inclusion to participants able to engage with the intervention so that safety and fidelity of digital health-based intervention administration can be established without technical confounders [68-70]. Future work will adapt the system for broader populations (multilingual versions, voice interfaces, assisted navigation) and test effectiveness in individuals with limited digital or health literacy.” (**Pages 36 -37; Lines 717 – 723**)

**iii) References (Citation order is as in the main manuscript)**

68. Marcolino MS, Oliveira JAQ, D’Agostino M, et al. The impact of mHealth interventions: systematic review of systematic reviews. *JMIR Mhealth Uhealth*. 2018;6(1):e23.

69. Mohr DC, Weingardt KR, Reddy M, Schueller SM. Three problems with current digital mental health research… and three things we can do about them. *Psychiatr Serv*. 2017;68(5):427–429.

70. Whittaker R, Merry S. Technology-dissemination and digital health intervention frameworks. *Lancet Digital Health*. 2021;3:e2–e3.

___________________________________________________________________________

**4) Reviewer’s Major Comment 4: Threshold for Unacceptable error rates**

While the manuscript outlines general plans for error logging and clinician review of 10% of sessions, it would benefit from specifying a threshold for unacceptable error rates that would trigger protocol revision or safety alerts.

**i) Author’s response:**

We thank the reviewer for this important suggestion. We agree that pre-defined thresholds are essential for transparent AI safety governance. In response, we have expanded the protocol to include a **quantitative, risk-based error taxonomy and escalation plan** with explicit safety triggers:

- **Error taxonomy (pre-specified):**
  - **Critical error:** AI output that could directly cause patient harm (e.g., incorrect dosing interval that risks electrolyte imbalance, or wrong instructions about fasting before sedation). Any critical error triggers **immediate suspension** of the chatbot for that pathway and urgent review by the trial Safety Officer and Data Monitoring Committee (DMC).
  - **Major error:** Incorrect advice that could reduce bowel preparation adequacy (e.g., instructing a patient to take the final laxative >12 hours before procedure). These require **prompt corrective action and expanded audit**.
  - **Minor error:** Non-clinical issues (typos, ambiguous phrasing) that do not affect safety or efficacy, but still require **content refinement** if recurrent.
- **Quantitative thresholds and actions (pre-specified):**
  - **Immediate suspension:** Any single **critical error** in an audited transcript will result in immediate suspension of the chatbot pathway and urgent DMC review.
  - **Prompt corrective action:** **Major errors ≥1%** of audited sessions in a rolling 2-week window for root-cause analysis, prompt correction, and expanded audit (25% sample) until rates fall below threshold.
  - **Content refinement:** **Minor errors ≥5%** across two consecutive audits will lead to content editing and template re-training, followed by monitoring.
- **Audit and monitoring schedule:**

Weekly automated error logging; clinician review of a random 10% of transcripts (escalated to 25% if thresholds are breached); and monthly safety summaries to the DMC. All transcripts contain version metadata, ensuring that any drift after model updates can be traced.

This approach is consistent with best practices from WHO [1], SPIRIT-AI/CONSORT-AI [2], BMJ safety frameworks [3], the Topol Review [4], NHSX policy [5], the NIST AI Risk Management Framework [6], and explainability scholarship in healthcare AI [7].

**ii) Exact Manuscript Changes:**

a) **“AI error taxonomy, thresholds and escalation**

All chatbot interactions will be logged and reviewed according to a pre-specified error taxonomy. Errors are classified as Critical, Major or Minor (definitions below). A weekly random sample of 10% of sessions will be reviewed by trained clinicians and review sampling will expand to 25% if thresholds are exceeded. Escalation rules are:

1. Critical error: any instance of advice that could cause direct physical harm (e.g., wrong dosing interval for the laxatives leading to electrolyte imbalance; instruction to discontinue essential medication without context) will immediately suspend the affected module and trigger urgent review by the Safety Officer and Data Monitoring Committee (DMC).

2. Major errors: defined as incorrect or misleading clinical advice that could materially reduce bowel preparation adequacy (e.g., wrong laxative timing exceeding 6 hours). If 1% or more of audited sessions in a rolling 2-week window contain Major errors, root cause analysis will be performed, chatbot prompts/content will be corrected, and audit sampling will increase to 25% until the error rate falls below the threshold.

3. Minor errors: non-material issues such as ambiguous wording, style, or formatting. If more than 5% of audited sessions contain minor errors in two consecutive reviews, content will be refined and monitored more closely.

This structured governance approach aligns with international AI safety recommendations, including WHO and SPIRIT-AI/CONSORT-AI guidance [29, 30], healthcare AI safety principles [31-33], the National Institute of Standards and Technology (NIST) AI Risk Management Framework [34], and explainability principles for multidisciplinary evaluation of AI in health [35].” (**Pages 19-20; Lines 285-307**)

**iii) References (Citation order is as in the main manuscript):**

29. World Health Organization. *Ethics and governance of artificial intelligence for health: WHO guidance*. Geneva: World Health Organization; 2021.

30. Rivera SC, Liu X, Chan AW, Denniston AK, Calvert MJ. CONSORT-AI and SPIRIT-AI: new reporting guidelines for clinical trials evaluating artificial intelligence interventions. *Lancet Digit Health*. 2020;2(10):e537–8.

31. Coiera E, Ammenwerth E, Georgiou A, Magrabi F. Safety of artificial intelligence in health care. *BMJ*. 2021;372:m693.

32. Topol EJ. *The Topol Review: Preparing the healthcare workforce to deliver the digital future*. NHS Health Education England; 2019.

34. NHSX. *Artificial Intelligence: How to get it right. Putting policy into practice for safe data-driven innovation in health and care*. London: NHSX; 2020.

35. National Institute of Standards and Technology. *AI Risk Management Framework 1.0*. Gaithersburg: NIST; 2023.

36. Amann J, Blasimme A, Vayena E, Frey D, Madai VI. Explainability for artificial intelligence in healthcare: a multidisciplinary perspective. *BMC Med Inform Decis Mak*. 2020;20(1):310.

___________________________________________________________________________

**5) Reviewer’s Major Comment 5: Objective Assessment of Adherence to Bowel Preparation Instructions**

It is unclear how adherence to bowel preparation instructions will be objectively assessed and compared between groups, especially since the chatbot allows for personalised interaction. Please define operational criteria for adherence in each arm (e.g., completion of laxative, compliance with timing).

**i) Author’s Response:**

We appreciate this demand for operational clarity. We now pre-specify adherence definitions, multiple sources of adherence data, and how adherence informs the per-protocol population. Our definitions are aligned with international guidance on bowel preparation adequacy [23,37], and our triangulated measurement/reporting framework follows recommendations for transparency in adherence and exposure assessment [38].

1. **Adherence definitions (pre-specified):**

• *Full adherence*: All prescribed sachets/doses consumed and the final dose started within 4-6 hours before procedure start and completed ≥2 hours before procedure start [23,37].

• *Partial adherence*: Single missed sachet/dose OR final dose completed outside the 2-6-hour window but within 12 hours.

• *Non-adherent*: >1 missed sachet/dose or final dose initiated >12 hours prior to procedure start or omitted.

2. **Measurement sources (triangulation):**

• Intervention arm (chatbot): Timestamped in-chat confirmations of dose completion, automatically logged in server logs.

• Control arm: Electronic diary entry (REDCap) or phone confirmation.

• All participants: Nurse-verified last oral intake on admission; discordant reports adjudicated.

3. **Use in analysis:**

The per-protocol (PP) population is defined as participants meeting Full adherence. Primary non-inferiority will be evaluated in both ITT and PP populations. Non-inferiority must be shown in both to be claimed.

These procedures increase objectivity, reduce bias from self-report, and allow a meaningful per-protocol analysis that is transparent and reproducible.

**ii) Exact Manuscript Changes:**

“**Assessments of Intervention Adherence**

Adherence will be assessed using a triangulated approach: (a) participant self-report with timestamps (intervention: chatbot confirmation logs; control: electronic diary/phone confirmation); (b) nurse-verified time of last oral intake on admission; and (c) cross-checking where discrepancies exist.

Adherence categories are pre-specified, consistent with ESGE guidelines and published adequacy thresholds [23,37]:

• Full adherence: all doses consumed and final dose initiated between 4 and 6 hours before procedure start and completed 2 hours or more before procedure.

• Partial adherence: Single missed dose or final dose outside the 2-to-6-hour window but within 12 hours.

• Non-adherent: More than one missed dose or final dose initiated more than 12 hours before procedure.

The per-protocol population comprises participants meeting full adherence. Adherence rates will be reported by arm and incorporated in sensitivity analyses, following recommendations for transparent adherence reporting [38].” (**Pages 22-23; Lines** **372-387**)

**iii) References (Citation order and numbering are as in the main manuscript):**

23. Hassan C, East J, Radaelli F, et al. Bowel preparation for colonoscopy: ESGE Guideline – Update 2019. *Endoscopy*. 2019;51(8):775–794.

37. Clark BT, Protiva P, Nagar A, et al. Quantification of adequate bowel preparation for screening or surveillance colonoscopy in men. *Gastroenterology*. 2016;150(2):396–404.e2.

38. Vandenbroucke JP, von Elm E, Altman DG, et al. Strengthening the Reporting of Observational Studies in Epidemiology (STROBE): explanation and elaboration. *Epidemiology*. 2007;18(6):805–835.

___________________________________________________________________________

**6) Reviewer’s Minor Comment 1: Inconsistency Between Superiority and Non-Inferiority Frameworks**

The manuscript inconsistently refers to BBPS as a non-inferiority outcome and sometimes as a superiority outcome. Please clarify this in both abstract and methods.

**i) Author’s Response**

Thank you for noting this inconsistency. We have standardized terminology throughout the manuscript: the trial is a **non-inferiority** randomized controlled trial and BBPS is the **sole primary endpoint** assessed in a non-inferiority framework. All instances of “superiority” have been removed or corrected. The Abstract and Methods (Sample Size and Statistical Analysis Plan (SAP) now consistently describe the one-sided non-inferiority hypothesis and the -1.1 margin.

**ii) Exact manuscript changes:**

**a) Abstract:** Replace “superiority” with “non-inferiority” in all locations in the abstract (**Page 2, Lines 29, 37-38 and 42**).

**b) Methods (In Statistical Analysis Subsection):** We ensure the paragraph specifies one-sided test α=0.025 and the non-inferiority hypotheses. (**Page 33; Lines 634**). All mention of superiority test and design framework have been removed.

___________________________________________________________________________

**7) Reviewer’s Minor Comment 2: Multiple mentions of SPIRIT AI Guideline in the Manuscript**

The manuscript refers to SPIRIT-AI in multiple sections in nearly identical wording. Consider reducing repetition for conciseness.

**i) Author’s Response:**

We thank the reviewer for this careful observation. On review, SPIRIT-AI was mentioned four times: once in the **Abstract**, once in the **Introduction**, and twice again in the **Discussion**. To avoid redundancy while still conveying compliance to the aforementioned reporting guidelines, we streamlined our manuscript as follows:

- **Abstract (Page 2, Lines 47):** We retained one concise sentence referring to SPIRIT 2025 and SPIRIT-AI, slightly polished for clarity.
- **Introduction (Page 6, Lines 128–129):** We retained a consolidated sentence that cites SPIRIT 2025 together with SPIRIT-AI and CONSORT-AI.
- **Discussion (Page 36, Lines 696–698):** We deleted two repeated sentences explicitly naming SPIRIT-AI. These were replaced with a single neutral summarizing sentence (“Besides, the trial design and reporting align with internationally recognized clinical trial standards, thereby supporting methodological rigor and reproducibility for AI-based interventions.”).

This reduces duplication, maintains transparency, and presents SPIRIT-AI alongside SPIRIT 2025 and CONSORT-AI in a balanced way.

___________________________________________________________________________

**B) Reviewer 2: Detailed Point-by-Point Responses**

**1) Reviewer’s Major Comment 1: Omission of Relevant ChatGPT-Colonoscopy Literature**

To assert in the Introduction that no data exist on the use of ChatGPT for bowel preparation in colonoscopy is unsustainable. A study has already been published that yielded preliminary results through a concordance analysis (<https://doi.org/10.3390/diagnostics14222537>). The authors have neither cited nor discussed this work, which is presently the only one indexed in MEDLINE. Although that study employed an LLM to evaluate the BBPS rather than to provide information, it should nevertheless be cited to demonstrate how ChatGPT recognizes the importance of bowel preparation for colonoscopy.

**i) Author’s Response**

We thank the reviewer for this correction. Our original wording in the Introduction was too absolute. We have now **cited and described Pellegrino et al. (Diagnostics, 2024)**, the only MEDLINE-indexed study to date that used ChatGPT-4 in the context of bowel preparation, and **clarified how it differs from our research question**.

**What Pellegrino et al. did and found was that** they conducted an **AI-to-image concordance analysis** in which ChatGPT-4 directly graded colonoscopy frames for bowel cleanliness using BBPS and was compared with human endoscopists. After exclusions, **587 images** were analyzed. ChatGPT-4 labelled **62.9%** of frames as adequately prepared (BBPS > 1) versus **89.3%** by humans, with **slight agreement** on adequacy (**κ = 0.099**) and a **lower median raw BBPS** by ChatGPT-4 [2 (IQR 1-3) vs human 3 (2-3)], with **moderate concordance** on raw scores (W = 0.554).

We now explain how our trial differs and why our study is relevant with direct reference to Pellegrino et al. Pellegrino et al. assessed whether a chatbot could grade colonoscopy images using the BBPS. Our protocol evaluates a patient-education adjunct (LLM-mediated counselling for bowel preparation) that uses ChatGPT as a large language model to improve adherence to standard bowel preparation instructions. The BBPS in our study is adjudicated only by masked endoscopists during colonoscopy, and the chatbot has no role in outcome scoring. We cite Pellegrino et al. to acknowledge prior ChatGPT-related work in this domain while clarifying that their study addresses diagnostic image scoring and that ours addresses a different question focused on patient education.

**ii) Exact manuscript changes (Citation order and numbering are as in the main manuscript):**

**a) Introduction**

“Recent work has examined ChatGPT-4 in a different part of the bowel-preparation pathway, namely, as a stand-alone image grader of bowel cleanliness on colonoscopy frames using the Boston Bowel Preparation Scale (BBPS). In that concordance analysis, ChatGPT-4 labelled fewer frames as adequately prepared than human endoscopists (62.9% vs 89.3%), with slight agreement on adequacy (κ = 0.099, p<0.001) and moderate concordance for raw BBPS scores (W = 0.554, p = 0.036) [18].” (**Page 5, Lines 91-96**)

**b) Discussion**

“Our findings will thus complement a recent AI-to-image concordance study in which ChatGPT-4 graded colonoscopy frames for BBPS and showed lower adequacy calls with slight agreement relative to expert raters [18]” (**Page 35, Lines 686-689**)

**iii) References (Citation order and numbering are as in the main manuscript):**

18) Pellegrino R, Federico A, Gravina AG. Conversational LLM Chatbot ChatGPT-4 for Colonoscopy Boston Bowel Preparation Scoring: An Artificial Intelligence-to-Head Concordance Analysis. *Diagnostics.* 2024;14(22):2537. doi:10.3390/diagnostics14222537.

___________________________________________________________________________

**2) Reviewer’s Major Comment 2: Inconsistency Between Superiority and Non-Inferiority Frameworks**

The study design and statistical plan describe a non-inferiority framework, yet the study is introduced as a “superiority randomised controlled trial”. This inconsistency must be corrected, and the primary hypothesis to be tested should be clearly defined.

**i) Author’s Response**

We agree that wording must be fully consistent with a non-inferiority framework. We have reviewed the manuscript and corrected any phrasing that could imply a superiority design. We already ensured that the manuscript **Title and Abstract** already describe a non-inferiority randomized controlled trial, and the **Methods section** lays out a non-inferiority analysis plan. These sections now read consistently across the paper. Evidence of the current framing appears in the Abstract and Methods text, where non-inferiority is specified and where the non-inferiority margin is defined in the confidence-interval statement.

We also make the primary hypothesis explicit to remove any ambiguity. The null hypothesis states that the intervention is worse than standard counselling by more than the prespecified non-inferiority margin of minus 1.1 BBPS points. The alternative hypothesis states that the difference between groups does not exceed minus 1.1 BBPS points. Non-inferiority will be concluded if the lower bound of the two-sided 95 percent confidence interval for the mean difference in total BBPS score, calculated as intervention minus control, is greater than -1.1. This statement follows guidance from the CONSORT extension for non-inferiority and equivalence trials [51].

**ii) Exact manuscript changes:**

**a) Abstract:** We replaced “superiority” with “non-inferiority” in all locations in the abstract (**Page 2, Lines 29, 37-38 and 42**).

**b) Methods in Statistical Analysis Subsection:** We ensured the paragraph specifies one-sided test α=0.025 and the non-inferiority hypotheses. (**Page 33; Lines 634**). All mention of superiority test and design framework have been removed.

**c)** “The primary non-inferiority analysis will use a one-sided t-test at an alpha level of 0.025, testing the null hypothesis that the mean difference in BBPS between the intervention and control groups is less than or equal to -1.1 against the alternative that it is greater than -1.1.” (**Page 31, Lines 592 – 595**)

**iii) References (Citation order and numbering are as in the main manuscript):**

51. Piaggio G, Elbourne DR, Pocock SJ, Evans SJW, Altman DG; CONSORT Group FT. Reporting of noninferiority and equivalence randomized trials. Extension of the CONSORT 2010 statement. JAMA. 2012;308(24):2594-2604. doi:10.1001/jama.2012.87802.

___________________________________________________________________________

**3) Reviewer’s Major Comment 3: Multiplicity issue for multiple primary endpoints**

The protocol lists BBPS, DASS-21 and PSQ-18 all as primary endpoints—three in total. How will multiplicity be handled? The resulting inflation of the alpha error will be considerable and must be discussed and justified; the sample-size calculation will presumably be greatly affected.

**i) Author’s response**

We thank the reviewer for highlighting this important statistical issue. We agree that presenting three co-primary endpoints, namely the Boston Bowel Preparation Score (BBPS), DASS-21 scores for anxiety evaluation, and PSQ-18 scores for assessing satisfaction, would inflate the family-wise type I error and complicate the interpretation of the trial results. In response, we have revised the outcome hierarchy and clarified the analysis strategy throughout our manuscript. The BBPS is now designated as the sole primary endpoint and will be analyzed for non-inferiority. This realignment of the analysis ensures that the statistical focus remains firmly on the trial’s core clinical objective, which is the objective quality of bowel preparation assessed by masked endoscopists during colonoscopy. By restricting the confirmatory hypothesis testing to BBPS, we avoid fragmentation of the pre-specified alpha error rate across multiple outcomes and eliminate the inflation of type I error.

This approach is consistent with accepted methodological and regulatory guidance, which strongly recommends that clinical trials pre-specify a single primary endpoint unless a compelling justification exists for multiple co-primary endpoints. Both the International Council for Harmonisation E9 guideline and the U.S. Food and Drug Administration’s guidance on multiple endpoints stress that an unambiguous primary endpoint is essential for confirmatory inference, since uncontrolled multiplicity across several endpoints risks undermining interpretability and regulatory acceptability. Our revision aligns with these principles and also reflects the arguments laid out in the statistical literature that describe how multiple primary hypotheses inflate type I error unless a multiplicity-control strategy such as Bonferroni, Holm, or hierarchical gatekeeping is implemented a priori [1–3].

Anxiety measured by DASS-21 and satisfaction measured by PSQ-18 remain valuable for capturing patient-centered outcomes, but they are now formally classified as secondary endpoints. These outcomes will be reported descriptively with effect estimates and two-sided 95% confidence intervals. Any p-values generated from these analyses will be regarded as exploratory and hypothesis-generating only, without multiplicity adjustment. This approach reflects standard practice when such outcomes are supportive rather than confirmatory. While we considered alternative options such as hierarchical testing, formal gatekeeping strategies, or multiplicity-adjusted procedures, these were rejected because BBPS is the only outcome intended to underpin the confirmatory non-inferiority conclusion. Furthermore, the subjective outcomes, although clinically important, were never intended to carry the inferential weight of the trial. Since the sample size was powered solely on BBPS, applying confirmatory testing procedures to additional endpoints would be statistically inappropriate and potentially misleading.

Finally, our sample size remains valid without recalculation because it was determined exclusively from the assumptions underpinning BBPS. The revised endpoint hierarchy therefore eliminates the risk of alpha inflation in the primary analysis, ensures that confirmatory inference rests only on the non-inferiority of BBPS, and still allows anxiety and satisfaction to be presented transparently as important but supportive secondary outcomes. These revisions bring the protocol into alignment with both methodological guidance and regulatory standards on multiplicity [1–3].

**ii) Exact manuscript changes**

**a) Abstract: “three primary endpoints” to “single primary and two secondary endpoints”**

“The primary endpoint is the BBPS, assessed for non-inferiority. Secondary endpoints are patient anxiety (DASS-21) and patient satisfaction assessed using DASS-21 and PSQ-18 questionnaires, respectively and the findings will be reported descriptively with two-sided 95% confidence interval and any p-values will be considered exploratory without multiplicity adjustment.” (**Page 2, Lines 37-41**)

**b) Statistical Analysis Plan**

“The primary analysis will then be performed using Analysis of Covariance (ANCOVA), adjusting for age, gender (male as the risk factor), prior colonoscopy experience, colonoscopy indication, and baseline constipation score for the primary trial outcome, BBPS [59-60]. This limited set of covariates was chosen to improve precision without overfitting, in line with evidence that excessive adjustment in small samples undermines model stability [61-62].” (**Page 31, Lines 582-587**)

“However, for all secondary trial endpoints, the findings will be considered supportive and exploratory only. The results will be summarized with two-sided 95% confidence intervals and any p-values will be treated as exploratory with no multiplicity adjustment, in line with guidance on multiplicity and endpoint hierarchy in clinical trials [52-53, 63].” (**Page 32, Lines 608-612**)

**c) Discussion**

“To avoid alpha-inflation, the protocol designates BBPS as the sole primary endpoint for confirmatory non-inferiority testing, whereas anxiety and satisfaction are secondary and interpreted descriptively [52-53, 63].” (**Page 35, Lines 684-686**)

**iii) References (Citation order and numbering are as in the main manuscript):**

52. International Conference on Harmonisation of Technical Requirements for Registration of Pharmaceuticals for Human Use. Statistical principles for clinical trials. ICH harmonised tripartite guideline E9. Step 4 version. Geneva: ICH; 1998 Feb. Available from: https://database.ich.org/sites/default/files/E9_Guideline.pdf. Date of access: 3rd Sep 2025.

53. U.S. Food and Drug Administration. Multiple Endpoints in Clinical Trials-Guidance for Industry. Silver Spring, MD: FDA; 2017. Available from: https://www.fda.gov/files/drugs/published/Multiple-Endpoints-in-Clinical-Trials-Guidance-for-Industry.pdf. Date of Access: 3rd September 2025.__

63. Dmitrienko A, D’Agostino R Sr. Multiplicity considerations in clinical trials. N Engl J Med. 2018;378(22):2115-2122. doi: 10.1056/NEJMra1709701.

____________________________________________________________________

**4) Reviewer’s Major Comment 4: The choice of 1.1 BBPS points Minimum Clinically Important Difference (MCID) for BBPS**

A two-sided test on a mean difference of 1.1 BBPS points is specified, but in a non-inferiority setting a one-sided test with H₀ defined on the delta margin is required. The delta of −1.1 should be based on solid clinical references (guidelines or correlation studies) and these should be cited, so that a loss of 1.1 points can be shown not to worsen the ADR in a clinically meaningful way, as well as remaining statistically coherent.

**i) Author’s Response**

We thank the reviewer for this valuable comment and completely agree that a non-inferiority trial must employ a one-sided hypothesis test anchored on a prespecified margin, rather than a two-sided framework. The Statistical Analysis section has therefore been revised to specify that the primary analysis will be conducted using a one-sided t-test at a type I error level of 0.025. The null hypothesis will state that the mean difference in BBPS between intervention and control groups is less than or equal to -1.1, with the alternative that the true difference exceeds -1.1. This ensures that the analysis is both statistically coherent and aligned with established principles of non-inferiority design.

Regarding the delta margin, we confirm that -1.1 BBPS points was chosen based on empirical validation studies and international guideline benchmarks, rather than arbitrarily. Lai and colleagues demonstrated in their validation of the BBPS that adequate preparation, defined as a total score of at least 6 with all segment scores ≥2, reliably predicts adenoma detection rates and caecal intubation rates [1]. Within this framework, they found that differences of approximately one point in the total BBPS did not alter the adequacy classification or compromise adenoma detection. Calderwood and Jacobson subsequently reinforced these findings in a comprehensive validation study, showing that small within-range changes did not significantly influence either caecal intubation success or adenoma detection rate [2]. Taken together, these studies confirm that a margin around -1.0 does not equate to a clinically meaningful deterioration of bowel preparation quality.

The European Society of Gastrointestinal Endoscopy (ESGE) guideline further supports this choice by defining adequacy benchmarks, recommending that at least 85% of colonoscopies should reach adequate preparation [3]. Even if the control mean BBPS were around 7.5, applying the non-inferiority margin of -1.1 would yield a mean of 6.4, which still preserves adequacy well above the 85% benchmark. The selected margin therefore balances statistical rigor with clinical conservatism, protecting interpretability of trial results without unnecessarily underpowering the study.

These revisions harmonize the statistical and clinical rationale for the non-inferiority framework and clarify the appropriate one-sided hypothesis test in the manuscript.

**ii) Exact Manuscript Changes**

“The non-inferiority margin of -1.1 BBPS points was selected based on validation studies showing that differences of this magnitude do not compromise adenoma detection rate or adequacy classification (BBPS ≥6 with all segments ≥2) [39, 40]. This threshold is also consistent with ESGE guideline quality benchmarks that require at least 85% adequacy in bowel preparation [25].” (**Page 31-32, Lines 596-600**)

**iii) References (Citation order and numbering are as in the main manuscript)**

25. Hassan C, East J, Radaelli F, Spada C, Benamouzig R, Bisschops R, et al. Bowel preparation for colonoscopy: European Society of Gastrointestinal Endoscopy (ESGE) Guideline – Update 2019. Endoscopy. 2019;51(8):775–94. doi:10.1055/a-0959-0505.

39. Lai EJ, Calderwood AH, Doros G, Fix OK, Jacobson BC. The Boston Bowel Preparation Scale: a valid and reliable instrument for colonoscopy-oriented research. Gastrointest Endosc. 2009;69(3 Pt 2):620–5. doi:10.1016/j.gie.2008.05.057.

40. Calderwood AH, Jacobson BC. Comprehensive validation of the Boston Bowel Preparation Scale. Gastrointest Endosc. 2010;72(4):686–92. doi:10.1016/j.gie.2010.06.068.

___________________________________________________________________________

**5) Reviewer’s Major Comment 5: Adequate power of the planned sample size for secondary trial endpoints**

Does the planned sample size retain adequate power for the secondary outcomes as well?

**i) Author’s Response**

We thank the reviewer for this important observation. We would like to clarify that the trial has been explicitly designed and powered only with respect to the primary endpoint, which is the Boston Bowel Preparation Scale (BBPS). The sample size calculation is based on the non-inferiority hypothesis concerning BBPS, using a one-sided test at α = 0.025 with a pre-specified non-inferiority margin of -1.1 points, as detailed in the Statistical Analysis section. The study is not powered to formally test hypotheses for secondary endpoints such as anxiety (DASS-21) or patient satisfaction (PSQ-18).

This decision reflects both methodological and regulatory best practice. As highlighted in the CONSORT extension for non-inferiority and equivalence trials and in methodological reviews of trial design by the International Council for Harmonisation (ICH) and US Food Drug Administration (FDA), non-inferiority trials should pre-specify a single primary endpoint that determines the required sample size, while secondary endpoints are usually assessed descriptively or with exploratory analyses [51, 52]. Attempting to power the trial for all secondary outcomes would require a prohibitively large sample size and would dilute the central inferential focus, which is the non-inferiority assessment of BBPS.

Accordingly, secondary outcomes will be reported with point estimates and 95% confidence intervals, and any associated p-values will be interpreted as exploratory rather than confirmatory. This ensures transparency while avoiding misleading inferences from underpowered hypothesis tests. Such an approach is consistent with international trial methodology standards and reporting guidelines, including the recommendations of Piaggio and co-workers [51], which emphasize the distinction between adequately powered primary analyses and descriptively interpreted secondary analyses in the context of non-inferiority trials.

We believe this strategy provides an appropriate balance between statistical rigor and feasibility, maintaining sufficient power for the clinically decisive endpoint (BBPS) while still generating valuable descriptive insights into patient-centered secondary outcomes.

**ii) Exact manuscript changes (In Methods, Sample Size Calculation)**

“The trial is only powered for the primary endpoint, the BBPS.” (**Page 26, Lines 469**)

“No formal sample size calculation was performed for anxiety or satisfaction scores due to limited prior data. The planned sample size is thus not powered to detect differences in secondary endpoints, specifically anxiety (DASS-21) and satisfaction (PSQ-18) scores. This approach is in line with the methodological standards for non-inferiority trials, in which the sample size is driven by a single clinically decisive primary endpoint, while secondary outcomes are used to provide supportive and contextual evidence [51-53].” (**Page 27, Lines 489-495**)

### iii) References (Citation order and numbering are as in the main manuscript):

51. Piaggio G, Elbourne DR, Pocock SJ, Evans SJW, Altman DG, CONSORT Group FT. Reporting of noninferiority and equivalence randomized trials: extension of the CONSORT 2010 statement. JAMA. 2012;308(24):2594–2604. doi:10.1001/jama.2012.87802.

52.International Conference on Harmonisation of Technical Requirements for Registration of Pharmaceuticals for Human Use. Statistical principles for clinical trials. ICH harmonised tripartite guideline E9. Step 4 version. Geneva: ICH; 1998 Feb. Available from: https://database.ich.org/sites/default/files/E9_Guideline.pdf. Date of access: 3rd Sep 2025.52.

53. U.S. Food and Drug Administration; Center for Drug Evaluation and Research; Center for Biologics Evaluation and Research. Multiple endpoints in clinical trials: guidance for industry. Draft guidance. Silver Spring (MD): US FDA; 2017 Jan. Available from: https://www.fda.gov/files/drugs/published/Multiple-Endpoints-in-Clinical-Trials-Guidance-for-Industry.pdf. Date of access: 3rd Sep 2025.

___________________________________________________________________________

**6) Reviewer’s Major Comment 6: ANCOVA and Number of Covariate Used for Statistical Adjustments**

The primary analysis proposes a t-test followed by a Mann–Whitney test, yet ANCOVA would be more appropriate, providing a 95% confidence interval to be compared with delta in a non-inferiority context. Moreover, with only 96 participants you intend to adjust for seven covariates, entailing a marked risk of over-fitting; likewise, imputing 20 datasets in so small a sample carries a high likelihood of spurious variance.

**i) Author’s Response**

We thank the reviewer for raising this important statistical concern regarding the risk of overfitting and the appropriate number of imputations in a trial with a modest sample size of 96 participants. We agree that the initial plan to adjust for seven covariates could create instability and inflate variance estimates in a relatively small dataset. Overfitting occurs when too many predictors are included relative to the available sample size, leading to biased regression coefficients, exaggerated standard errors, and spurious associations that do not generalize beyond the study sample. This phenomenon has been extensively described in the methodological literature. For instance, Peduzzi and colleagues demonstrated in the context of logistic regression that when the ratio of events per variable falls below ten, parameter estimates become unreliable and confidence intervals widen disproportionately [61]. Similarly, Babyak provided a broad review of overfitting in regression analyses in clinical research, emphasizing that an excessive number of covariates relative to the sample size increases the risk of unstable models and invalid inference [62].

In line with this body of evidence, we have reduced the number of adjustment covariates from seven to five, retaining only those with the strongest clinical and methodological justification: age, sex, prior colonoscopy, indication for colonoscopy, and baseline constipation score. This strategy ensures that the model gains precision through covariate adjustment without introducing undue instability. Importantly, the chosen covariates are routinely recommended as key confounders in colonoscopy trials and are clinically interpretable. By limiting the adjustment set to five variables, the participant-to-predictor ratio remains acceptable, mitigating the reviewer’s concern regarding overfitting while preserving clinical relevance.

Regarding the imputation strategy, we agree that creating twenty imputed datasets in a sample of 96 could produce excessive between-imputation variance, leading to spurious results. The purpose of multiple imputation is to reduce bias and improve efficiency by accounting for uncertainty due to missing data, but this must be balanced with sample size. Rubin’s foundational work on multiple imputation established the principle that the number of imputations should be sufficient to achieve stable estimates of between-imputation variance but need not be excessive when the sample is small [56]. More recent practical guidance by White and co-workers in **Statistics in Medicine** suggests that while more imputations are beneficial with larger samples or high proportions of missing data, for smaller datasets a lower number of imputations (for example, five) is often adequate and avoids artificial inflation of variance estimates [57]. Jakobsen et al. further reinforced that multiple imputation should be tailored to the trial size and context, warning against unnecessarily large numbers of imputations in small clinical studies [58].

Taking these considerations into account, we will now perform multiple imputation with **m = 5** datasets, which is both statistically coherent and proportionate to the trial size. This number provides sufficient stability of variance estimates without introducing the distortions that may arise from over-imputation in a small sample. To further reassure the robustness of our findings, we will conduct sensitivity analyses, including complete-case analysis and worst-case imputation. These complementary approaches will allow us to evaluate whether the primary findings are sensitive to the chosen imputation strategy, thereby strengthening the reliability of our conclusions.

In summary, the revisions address both of the reviewer’s concerns. By reducing the covariate set from seven to five, we substantially reduce the risk of model overfitting while preserving essential clinical adjustment. By limiting imputations to five datasets, we align with best practice for small-sample trials, supported by both foundational and modern methodological authorities. We believe these changes enhance the statistical validity of the planned analysis while remaining transparent and methodologically rigorous.

**ii) Exact Manuscript Changes (in Statistical Analysis Subsection):**

“Following recommended practice, five imputed datasets will be generated, which is considered appropriate for this trial size and avoids spurious variance inflation [56-58]. (**Page 31, Lines 576-578**)

“The primary analysis will then be performed using Analysis of Covariance (ANCOVA), adjusting for age, sex, prior colonoscopy experience, colonoscopy indication, and baseline constipation score for the primary trial outcome, BBPS [59-60]. This limited set of covariates was chosen to improve precision without overfitting, in line with evidence that excessive adjustment in small samples undermines model stability [61-62].” **(Page 31, Lines 582-587**)

**iii) References (Citation order and numbering are as in the main manuscript):**

56. Little RJ, Rubin DB. Statistical Analysis with Missing Data. 3rd ed. Hoboken, NJ: John Wiley & Sons; 2019.

57. White IR, Royston P, Wood AM. Multiple imputation using chained equations: issues and guidance for practice. Stat Med. 2011;30(4):377–399. doi:10.1002/sim.4067

58. Jakobsen JC, Gluud C, Wetterslev J, Winkel P. When and how should multiple imputation be used for handling missing data in randomised clinical trials – a practical guide with flowcharts. BMC Med Res Methodol. 2017;17(1):162. doi:10.1186/s12874-017-0442-1

61. Peduzzi P, Concato J, Kemper E, Holford TR, Feinstein AR. A simulation study of the number of events per variable in logistic regression analysis. J Clin Epidemiol. 1996;49(12):1373–1379. doi:10.1016/S0895-4356(96)00236-3

62. Babyak MA. What you see may not be what you get: a brief, nontechnical introduction to overfitting in regression-type models. Psychosom Med. 2004;66(3):411–421. doi:10.1097/01.psy.0000127692.23278.a9

___________________________________________________________________________

**7) Reviewer’s Major Comment 7: Timing and Sedation Effects on DASS-21 and PSQ-18**

The DASS-21 measures “sub-acute” anxiety and thus does not capture the immediate anxiety on the day of the examination, as it refers to the preceding week; similarly, the PSQ-18 is administered post-procedure, so post-sedative symptoms may influence it. These limitations need to be discussed.

**i) Author’s Response:**
We thank the reviewer for this valuable comment concerning the temporal specificity of the DASS-21 and the potential for sedative effects to influence PSQ-18 scores. While we acknowledge these points, we respectfully disagree with the assertion that DASS-21 is unsuitable in this peri-procedural context. Although the standard recall period of DASS-21 is one week, the scale has demonstrated sensitivity to fluctuations within that timeframe [1-3] and correlates strongly with state anxiety measures such as the State-Trait Anxiety Inventory (STAI) [1]. For instance, Henry and Crawford reported that DASS-21 anxiety scores were responsive to situational stressors in non-clinical populations [3], while subsequent studies have confirmed its validity in different races and clinical contexts [4,5]. Moreover, research in gastrointestinal endoscopy [6-7], general cancer patients and oncology patients receiving chemotherapy (a subset of the study participants for the latter two studies includes colorectal cancer patients [8-9]) has demonstrated that DASS-21 correlates with immediate pre-procedural anxiety and distress, supporting its pragmatic use in this trial setting. These findings indicate that although DASS-21 is not a real-time “moment-to-moment” measure of anxiety, it remains a valid and feasible tool for assessing peri-procedural psychological states in a clinical trial context.

That said, we accept that DASS-21 may not fully capture acute spikes in anxiety occurring specifically on the day of colonoscopy. We will explicitly acknowledge this limitation in the manuscript to ensure clarity of interpretation.

With respect to the PSQ-18, we agree that residual sedative effects may influence patient satisfaction scores when the instrument is administered shortly after colonoscopy. This issue, however, is a common and well-recognized feature of satisfaction measurement in endoscopy where sedation is routine. Importantly, PSQ-18 and its derivatives have been validated in gastrointestinal populations undergoing sedation, including colonoscopy, and have consistently demonstrated reliability and construct validity [10–13]. For example, Ko et al. (2015) showed that patient-reported satisfaction in colonoscopy remained robust despite varying sedation levels [13]. As both groups in our trial will undergo similar sedation protocols, any sedative-related influence is expected to be non-differential and therefore should not bias between-group comparisons.

In summary, while both DASS-21 and PSQ-18 have limitations relating to temporal specificity and sedation effects, respectively, they remain validated, widely used, and clinically meaningful measures. Their use provides valuable insight into peri-procedural anxiety and satisfaction in the context of this randomized controlled trial, and we have revised the manuscript to explicitly acknowledge these considerations.

**ii) Exact manuscript changes**

“The DASS-21 and PSQ-18 were selected since both have been validated and widely used instruments with strong psychometric properties in our setting. We acknowledge, however, that DASS-21 uses a one-week recall frame and may not fully capture very acute anxiety fluctuations on the day of colonoscopy and that PSQ-18 scores could be influenced by residual sedation. We will acknowledge this limitation when interpreting the trial results, though the application of identical instruments across both groups may reduce the risk of bias.” **(Page 24, Lines 408-415).**

**References Used for Rebuttal:**

1. Antony MM, Bieling PJ, Cox BJ, Enns MW, Swinson RP. Psychometric properties of the 42-item and 21-item versions of the Depression Anxiety Stress Scales in clinical groups and a community sample. Psychol Assess. 1998;10(2):176-181. doi:10.1037/1040-3590.10.2.176

2. Taouk M, Lovibond PF, Laube R. Psychometric properties of an Arabic version of the Depression Anxiety Stress Scales (DASS21). Psychol Assess. 2001;13(2):186-193. doi:10.1037/1040-3590.13.2.186

3. Henry JD, Crawford JR. The short-form version of the Depression Anxiety Stress Scales (DASS-21): Construct validity and normative data in a large non-clinical sample. Br J Clin Psychol. 2005;44(Pt 2):227-239. doi:10.1348/014466505X29657

4. Norton PJ. Depression Anxiety and Stress Scales (DASS-21): Psychometric analysis across four racial groups. Anxiety Stress Coping. 2007;20(3):253-265. doi:10.1080/10615800701309279

5. Ng F, Trauer T, Dodd S, Callaly T, Campbell S, Berk M. The validity of the 21-item version of the Depression Anxiety Stress Scales as a routine clinical outcome measure. Acta Neuropsychiatr. 2007;19(5):304-310. doi:10.1111/j.1601-5215.2007.00217.x

6. Alam RR, Elashri NIE. Effectiveness of nursing interventions on level of anxiety, stress, and depression of elderly patients undergoing upper gastrointestinal endoscopy. Int J Adv Nurs Stud. 2020;9(1):20-28. doi:10.14419/ijans.v9i1.30560.

7. Tshabalala SJ, Tomita A, Ramlall S. Depression, anxiety and stress symptoms in patients presenting with dyspepsia at a regional hospital in KwaZulu-Natal province. S Afr J Psychiatr. 2019;25:1382. doi: 10.4102/sajpsychiatry.v25i0.1382

8. Fox RS, Lillis TA, Gerhart J, Hoerger M, Duberstein P. Multiple Group Confirmatory Factor Analysis of the DASS-21 Depression and Anxiety Scales: How Do They Perform in a Cancer Sample? Psychol Rep. 2018;121(3):548-565. doi: 10.1177/0033294117727747.

9. McMullen M, Lau PKH, Taylor S, McTigue J, Cook A, Bamblett M, Hasani A, Johnson CE. Factors associated with psychological distress amongst outpatient chemotherapy patients: An analysis of depression, anxiety and stress using the DASS-21. Appl Nurs Res. 2018;40:45-50. doi: 10.1016/j.apnr.2017.12.002.

10. Cheung YB, Ong YT, Thumboo J, Gao F, Ng WY. Assessment of depression, anxiety and quality of life in patients with colorectal cancer using the DASS-21 and EQ-5D. Support Care Cancer. 2015;23(8):2361-2370. doi:10.1007/s00520-014-2596-7

11. Marshall GN, Hays RD. The Patient Satisfaction Questionnaire Short-Form (PSQ-18). Santa Monica, CA: RAND Corporation; 1994.

12. Bjertnaes OA, Sjetne IS, Iversen HH. Overall patient satisfaction with hospitals: Effects of patient-reported experiences and fulfilment of expectations. BMJ Qual Saf. 2012;21(1):39-46. doi:10.1136/bmjqs-2011-000137

13. Ko HH, Zhang H, Telford JJ, Enns R. Factors influencing patient satisfaction when undergoing endoscopic procedures. Gastrointest Endosc. 2015;81(4):1034-1040. doi:10.1016/j.gie.2014.09.009

**___________________________________________________________________________**

**8) Reviewer’s Major Comment 8: Exclusion of patients with clinical anxiety**

Why exclude patients with clinical anxiety? Anxiety is an endpoint, and such patients are precisely those who might benefit from the intervention you propose.

**i) Author’s Response:**

We thank the reviewer for raising this important point regarding our exclusion of patients with clinically diagnosed anxiety disorders. While we recognize that such patients may have elevated procedural anxiety and could potentially benefit from the intervention, their exclusion was a deliberate methodological safeguard.

First, anxiety arising in patients with formal psychiatric diagnoses is distinct from situational or procedural anxiety. Chronic disorders such as generalized anxiety disorder and panic disorder are characterized by trait-level anxiety, maladaptive cognitive schemas, and chronic symptom trajectories that extend beyond the acute stressor of colonoscopy [1]. Including these patients would make it difficult to disentangle the specific effect of the intervention on transient, situational anxiety from the background influence of long-standing psychiatric morbidity.

Second, pharmacological treatment represents a major confounder. Patients with clinical anxiety commonly receive benzodiazepines, Selective Serotonin Reuptake Inhibitors (SSRIs), Serotonin-Norepinephrine Reuptake Inhibitors (SNRIs), or tricyclic antidepressants. Benzodiazepines exert immediate anxiolytic effects, directly altering DASS-21 and PSQ-18 outcomes, while antidepressants modulate affective reporting more gradually but still influence anxiety measures [2,3]. Such pharmacotherapy would create heterogeneity and introduce treatment-outcome interactions that cannot be reliably adjusted for, especially in a modestly sized trial.

Third, inclusion of patients with psychiatric disorders in a small trial (n=96) carries a significant risk of arm imbalance. Even a handful of such patients distributed unequally could disproportionately influence mean anxiety scores, obscure intervention effects, or generate spurious findings. In this setting, exclusion was necessary to protect internal validity and ensure the interpretability of results.

Fourth, this exclusion criterion is consistent with established methodological precedent. Peri-procedural anxiety trials, including those validating the State-Trait Anxiety Inventory (STAI), routinely exclude patients with psychiatric diagnoses to maintain homogeneity [4,5]. In gastroenterology, several colonoscopy anxiety studies have been done in the same fashion. Ko and co-workers excluded patients with psychiatric disorders when evaluating music therapy for colonoscopy [6]. Ovayolu and colleagues excluded patients with ongoing psychiatric treatment in their RCT comparing music and midazolam [7]. More recently, Veldhuijzen and co-workers conducted a multicenter RCT comparing computer-based education with nurse counselling before colonoscopy (CBE vs nurse counseling). Their **exclusion criteria** included “audiovisual or mental disabilities” (i.e., excluding psychiatric or cognitive impairment) [8]. These precedents demonstrate that our exclusion aligns with best practice in gastroenterology research.

Finally, we recognize that this decision limits external validity. Our findings cannot be directly generalized to patients with baseline psychiatric disorders, who may represent a distinct subgroup with different trajectories of peri-procedural anxiety. We have revised the manuscript to explicitly acknowledge this limitation. We also agree that future stratified or dedicated studies are warranted to determine whether non-pharmacological peri-procedural interventions confer benefit in this clinically important population.

Taken together, the exclusion of patients with clinical anxiety disorders was necessary to safeguard internal validity, reduce pharmacological confounding, and prevent imbalance in this small-sample trial, while still adhering to methodological precedent in the field.

**ii) Exact Manuscript Changes:**

“Third, we excluded participants with diagnosed clinical anxiety disorders to minimize confounding from psychiatric comorbidities and concurrent pharmacotherapy since both of which could mask or exaggerate the intervention’s effect on situational procedural anxiety, thus limiting the generalizability of our trial findings. Given the modest sample size, inclusion of such patients also risked an imbalance between study arms that could distort outcomes. This approach is consistent with methodological practice in peri-procedural anxiety research, including colonoscopy studies, but it limits applicability to patients with baseline psychiatric diagnoses. Future studies should address this subgroup specifically, as they may derive distinct benefits from tailored interventions.” **(Page 37, Lines 724-733)**

**iii) References Used for Rebuttal:**

1. Bandelow B, Michaelis S. Epidemiology of anxiety disorders in the 21st century. Dialogues Clin Neurosci. 2015;17(3):327-335. doi:10.31887/DCNS.2015.17.3/bbandelow

2. Baldwin DS, Waldman S, Allgulander C. Evidence-based pharmacological treatment of generalized anxiety disorder. Int J Neuropsychopharmacol. 2011;14(5):697-710. doi:10.1017/S1461145710001434

3. Offidani E, Guidi J, Tomba E, Fava GA. Efficacy and tolerability of benzodiazepines versus antidepressants in anxiety disorders: a systematic review and meta-analysis. Psychother Psychosom. 2013;82(6):355-362. doi:10.1159/000353198

4. Spielberger CD, Gorsuch RL, Lushene RE. Manual for the State-Trait Anxiety Inventory. Palo Alto, CA: Consulting Psychologists Press; 1970.

5. Millar K, Jelicic M, Bonke B, Asbury AJ. Assessment of preoperative anxiety: comparison of measures in patients awaiting surgery for breast cancer. Br J Anaesth. 1995;74(2):180-183. doi:10.1093/bja/74.2.180

6. Ko SY, Leung DY, Wong EM. Effects of easy listening music intervention on satisfaction, anxiety, and pain in patients undergoing colonoscopy: a pilot randomized controlled trial. Clin Interv Aging. 2019;14:977-986. doi: 10.2147/CIA.S207191.

7. Ovayolu N, Ucan O, Pehlivan S, Pehlivan Y, Buyukhatipoglu H, Savas MC, Gulsen MT. Listening to Turkish classical music decreases patients' anxiety, pain, dissatisfaction and the dose of sedative and analgesic drugs during colonoscopy: a prospective randomized controlled trial. World J Gastroenterol. 2006;12(46):7532-7536. doi: 10.3748/wjg.v12.i46.7532.

8. Veldhuijzen G, Klemt-Kropp M, sive Droste JS, van Balkom B, van Esch AA, Drenth JP. Computer-based patient education is non-inferior to nurse counselling prior to colonoscopy: a multicenter randomized controlled trial. Endoscopy. 2021;53(03):254-63. doi: http://dx.doi.org/10.1055/a-1225-8708

__________________________________________________________________________

**9) Reviewer’s Major Comment 9: ChatGPT as a non-open source AI technology**

ChatGPT is not open source, contrary to the statement made in the manuscript.

**i) Author’s Response:**

We thank the reviewer for pointing out this important factual inaccuracy. We fully agree that ChatGPT is not an open-source platform, and we regret the earlier imprecise wording that may have conveyed this impression. ChatGPT is in fact a proprietary large language model developed and maintained by OpenAI. Its parameters, training data, and underlying architecture are not publicly available in an open-source form, although certain research outputs and smaller models from the developer have been shared with the academic community.

In the context of our trial, this distinction does not alter the scientific integrity of the intervention or the methodological validity of our design. The version of ChatGPT that we integrated into our intervention is commercially available and accessible to healthcare providers, patients, and researchers under license. Therefore, our intervention reflects a real-world, pragmatic application of a widely used proprietary tool, rather than a bespoke or inaccessible system.

We have revised the manuscript to ensure clarity and transparency. The corrected statement now reads: *“…a proprietary large language model developed by OpenAI”* instead of the original phrase *“open-source”* throughout the manuscript. This correction ensures that our description of the intervention is factually accurate and avoids any potential misinterpretation by readers.

**ii) Exact Manuscript Changes (in Method and Discussion sections):**

a) “Since ChatGPT is a proprietary model….” (**Page 20, Lines 312-313**)

b) “ChatGPT 4.0 is a proprietary model not available for modification or distribution.” (**Page 22, Line 364**)

c) “Besides, the ChatGPT utilized in this study is a proprietary large language model…” (**Page 25, Line 445**)

___________________________________________________________________________

**10) Reviewer’s Major Comment 10: Feasibility of Patient Recruitment**

Is it feasible to recruit patients meeting all these criteria within a few months without adopting a multicentre design?

**1) Author’s response:**

We appreciate the reviewer’s concern regarding recruitment feasibility. We have carefully examined this issue using both our institutional colonoscopy volume data and prior recruitment experience from randomized controlled trials conducted in our endoscopy unit. On average, approximately 250 colonoscopies are performed each month at our center. Based on historical audits and screening logs, we estimate that around 40 percent of these patients would meet our eligibility criteria after applying exclusions, including those related to procedural and psychiatric factors. Of these eligible patients, we anticipate a conservative consent rate of approximately 50 percent, consistent with previous recruitment patterns in trials conducted at our institution.

Using these assumptions, we would expect roughly 100 potentially eligible patients per month, yielding about 50 enrolled participants. This means that the target of 96 participants can reasonably be achieved within a 3-month window in a single-center design. This estimation is intentionally conservative, as prior studies in our unit have sometimes achieved higher consent rates when study interventions were perceived as low-burden and patient-centered.

We also recognize that recruitment feasibility is a common concern in single-center studies. To mitigate risk, we have put contingency plans in place, including close monitoring of recruitment progress, weekly reporting to the trial steering group, and the option of extending the recruitment window if necessary. We believe these safeguards, combined with our strong historical track record of achieving recruitment targets for endoscopy-related trials, support the feasibility of the proposed design without requiring a multicenter approach.

**ii) Exact manuscript changes:**

“Based on our institutional audit data (around 250 colonoscopies per month, with approximately 40 percent meeting eligibility and a 50 percent consent rate), we expect to recruit 96 participants within 3 months (maximum 5 months if needed) at a single center. Recruitment progress will be monitored weekly and contingency strategies are in place should accrual rates fall below expectations based on this traffic light criteria:” (**Page 28, Lines 503-507**)

___________________________________________________________________________

**11) Reviewer’s Major Comment 11: The utilization of oncological CTCAE grading system for diagnostic colonoscopies**

Why employ an oncological CTCAE for diagnostic colonoscopies?

**1) Author’s response**

We appreciate the opportunity to clarify our safety reporting framework. CTCAE v5.0 provides a mature, widely used terminology with explicit, ordinal severity grades that support consistent AE capture and between-study comparability. Although CTCAE originated in oncology, the document is a general adverse-event terminology and grading system and is not restricted to cancer contexts [49]. We will apply only items relevant to colonoscopy and the peri-procedural period, for example, gastrointestinal hemorrhage, perforation, abdominal pain, nausea or vomiting, hypotension and hypoxia. To align with gastroenterology practice and maximize interpretability for the endoscopy community, we will also classify AE types using the American Society of Gastrointestinal Endoscopy (ASGE) endoscopic adverse-event lexicon [47] and will report rates in the format recommended by ASGE guidance on colonoscopy complications [48]. This dual approach preserves the field’s preferred nomenclature while retaining CTCAE’s graded severity scale for transparent cross-trial comparison.

In our previous draft, we inadvertently omitted explicit mention of the ASGE lexicon and reporting framework. We have now corrected this. At our academic teaching hospital, AE capture according to ASGE definitions is standard practice and is routinely documented by colonoscopists/endoscopists. This ensures methodological rigor and alignment with current gastroenterology standards.

**ii) Exact manuscript changes**

“AEs related to colonoscopies will be classified using the American Society for Gastrointestinal Endoscopy (ASGE) endoscopic adverse-event lexicon [47] and reported according to ASGE guidance for colonoscopy complications [48]. Severity of AEs will be classified based on the Common Terminology Criteria for Adverse Events (CTCAE) Version 5 [49], restricted to items relevant to colonoscopy and the peri-procedural period, including gastrointestinal bleeding, perforation, abdominal pain, nausea or vomiting, hypotension and hypoxia. This combined framework combines field-standard endoscopy terminology with a widely used graded severity scale to improve consistency, transparency and comparability across studies.” (**Page 26, Lines 452-461**)

**iii) References (Citation order and numbering are as in the main manuscript):**

47. Cotton PB, Eisen GM, Aabakken L, Baron TH, Hutter MM, Jacobson BC, et al. A lexicon for endoscopic adverse events: report of an ASGE workshop. Gastrointest Endosc. 2010;71(3):446-454. doi:10.1016/j.gie.2009.10.027

48. Fisher DA, Maple JT, Ben-Menachem T, Cash BD, Decker GA, Early DS, et al. Complications of colonoscopy. Gastrointest Endosc. 2011;74(4):745-752. doi:10.1016/j.gie.2011.07.025

49. U.S. Department of Health and Human Services, National Institutes of Health, National Cancer Institute. Common Terminology Criteria for Adverse Events (CTCAE) Version 5.0. Bethesda, MD: National Cancer Institute; 2017 Nov. Available from:https://ctep.cancer.gov/protocoldevelopment/electronic_applications/docs/CTCAE_v5_Quick_Reference_8.5x11.pdf. Date of access: 27th May 2025.

__________________________________________________________________________

**C) Reviewer 3: Detailed Point-by-Point Responses**

**1) Reviewer’s Major Comment 1: Eligibility Criteria and Potential Confounding by Previous Colonoscopy**

One aspect of your eligibility criteria allows both first-time and repeat elective colonoscopy patients to enrol. Patients who have previously undergone colonoscopy may already be familiar with dietary restrictions and bowel-preparation instructions, potentially affecting their baseline knowledge, anxiety and adherence compared with first-time patients. How do you plan to account for this difference in your study design or analysis? Have you considered stratifying randomisation by prior colonoscopy experience or adjusting for it in your statistical models to ensure that the intervention’s effect is not confounded by participants’ prior familiarity with the procedure?

**i) Author’s Response**

We thank the reviewer for this insightful comment regarding the potential influence of prior colonoscopy experience. We agree that repeat patients may differ from first-time patients in terms of baseline knowledge of bowel preparation protocols, perceived anxiety and procedural adherence. Although stratification by prior colonoscopy experience was not implemented because randomization was conducted using a simple sequence, we have incorporated analytic safeguards to address this potential confounding factor.

Prior colonoscopy status is systematically captured in the case report form for all participants. This variable is included as a pre-specified covariate in the ANCOVA model for the primary endpoint (BBPS), ensuring that any imbalances between first-time and repeat patients are adjusted for in the primary analysis [64]. In addition, exploratory subgroup analyses will compare outcomes separately for first-time and repeat patients, with a treatment-by-prior-colonoscopy interaction term assessed descriptively to evaluate consistency of intervention effects. While we acknowledge that subgroup analyses are underpowered for confirmatory inference, they provide transparency and reassurance about the robustness of results. This dual strategy of covariate adjustment and descriptive subgroup reporting is consistent with methodological recommendations for handling baseline heterogeneity in randomized trials [65].

These measures ensure that the influence of prior colonoscopy experience is carefully addressed in the analysis stage, despite the absence of stratification. Importantly, these analytic approaches were pre-specified in the protocol prior to initiation of data collection, and therefore represent proactive planning rather than post hoc modification.

**ii) Exact manuscript changes**

**a) Methods: In Statistical Analysis**

“The primary analysis will then be performed using Analysis of Covariance (ANCOVA), adjusting for age, sex, prior colonoscopy experience, colonoscopy indication, and baseline constipation score for the primary trial outcome, BBPS [59-60].” (**Page 31, Lines 582-585**)

**b) Case Report Form Variables:**

*“*

| Prior Colonoscopy History (if yes, please provide the year of most recent colonoscopy) | Yes/No  Year of most recent colonoscopy: |
| --- | --- |

*”* (**Table 3 (Row 11), Page 11**)

**c) Discussion**

“Finally, we acknowledge that both first-time and repeat patients will be enrolled under simple randomization without a stratification scheme, hence introducing some degree of bias due to prior participants’ prior colonoscopy experience. We will address this analytically by including it as a covariate and reporting subgroup analyses. This approach mitigates confounding risk, though a small degree of residual imbalance cannot be entirely excluded [64, 65].” (**Page 38, Lines 745-750**)

**iii) References (Citation order and numbering are as in the main manuscript):**

64. Vickers AJ, Altman DG. Statistics notes: Analysing controlled trials with baseline and follow up measurements. *BMJ*. 2001;323(7321):1123–4. doi:10.1136/bmj.323.7321.1123.

65. Brookes ST, Whitely E, Egger M, Smith GD, Mulheran PA, Peters TJ. Subgroup analyses in randomized trials: risks of subgroup-specific analyses; power and sample size for the interaction test. *J Clin Epidemiol*. 2004;57(3):229–36. doi:10.1016/j.jclinepi.2003.08.009.

___________________________________________________________________________

**2) Reviewer’s Major Comments 2: The number of endoscopists involved for BBPS scoring and any training or calibration procedure to ensure the consistency of BBPS scoring**

The protocol indicates that outcome assessments will be performed by experienced endoscopists who are blinded to the intervention. However, the manuscript does not specify how many endoscopists will participate in the trial or how inter‑observer variability will be managed. Could you clarify the number of endoscopists involved and describe any training or calibration procedures to ensure consistent scoring of the BBPS?

**i) Authors responses:**

We thank the reviewer for this valuable comment regarding outcome assessment reliability. We agree that inter-observer variability in BBPS scoring is an important methodological concern that must be addressed explicitly to ensure the validity and reproducibility of results.

In this trial, colonoscopies will be performed and scored by six consultant-level endoscopists, each with over five years of independent practice. Prior to trial commencement, the endoscopists underwent a structured calibration exercise using recorded colonoscopy videos, which were scored independently and then reviewed in consensus meetings until concordance was achieved. This procedure has been demonstrated to improve reproducibility in BBPS application [39,40].

During the trial, segmental BBPS scoring will be performed immediately after withdrawal to minimize recall bias. To maintain ongoing reliability, 10% of procedures will be randomly selected for secondary review of images by another blinded endoscopist. The agreement will be quantified using intra-class correlation coefficients (ICC), consistent with validation studies of the BBPS [39, 40]. Periodic calibration meetings will also be held to discuss discrepancies and reinforce scoring uniformity.

Furthermore, as part of the statistical analysis plan, ICC values will be reported descriptively as secondary quality-control outcomes. This ensures transparency in reliability assessment and provides readers with quantifiable evidence of the robustness of the scoring process.

**ii) Exact manuscript changes**

**a) Methods: In Trial Endpoint Assessments**

“Colonoscopy and BBPS scoring will be performed by two consultant-level endoscopists, each with at least five years of independent colonoscopy practice and have carried out at least 200 colonoscopies per year. Prior to trial commencement, all participating endoscopists undertook a structured calibration exercise involving independent scoring of recorded colonoscopy videos using the BBPS, followed by consensus discussion until concordance was reached. During the trial, inter-observer reliability will be evaluated by secondary review of 10% of procedures using recorded images, with agreement quantified using intra-class correlation coefficients (ICC). Periodic calibration meetings will be held to maintain consistency [39, 40].” (**Page 23, Lines 393-401**)

**b) Methods: In Statistical Analysis Plan**

“Besides, as a measure of quality control, inter-observer reliability for BBPS scoring will also be reported using intra-class correlation coefficients (ICC) derived from the 10% of procedures undergoing dual review [39]. These results will be presented descriptively as secondary trial outcomes.” (**Page 32, Lines 614-617**)

**iii) References (Citation order and numbering are as in the main manuscript)**

39. Lai EJ, Calderwood AH, Doros G, Fix OK, Jacobson BC. The Boston Bowel Preparation Scale: a valid and reliable instrument for colonoscopy-oriented research. Gastrointest Endosc. 2009;69(3 Pt 2):620-625. doi:10.1016/j.gie.2008.05.057.

40. Calderwood AH, Schroy PC 3rd, Lieberman DA, Logan JR, Zurfluh M, Jacobson BC. Boston Bowel Preparation Scale scores provide a standardized definition of adequate for describing bowel cleanliness. Gastrointest Endosc. 2014;80(2):269-276. doi:10.1016/j.gie.2014.01.031.

___________________________________________________________________________

**3) Reviewer’s Major Comment 3: Protocol Differences in Laxative Timing and Dosage Compared with ESGE Guidelines**

The chatbot’s content is based on guidelines from ESGE and MOH. However, the laxative timing and dosage in your protocol are slightly different from the 2019 ESGE recommendations. Could you specify which year/version of the ESGE guidelines you followed and explain any deviations in timing or dosage? How did you ensure that your regimen remains evidence-based given these differences?

**i) Authors’ Response:**

We thank the reviewer for this constructive comment regarding the alignment of our bowel preparation regimen with international guidance. The chatbot educational content and protocol regimen were developed in reference to the 2019 European Society of Gastrointestinal Endoscopy (ESGE) Guideline Update on bowel preparation for colonoscopy [24], in combination with the Malaysian Ministry of Health (MOH) Clinical Practice Guidelines on colorectal cancer screening (2017 edition) [25].

The principal difference is that while ESGE recommends split-dose regimens with the last dose administered 4 to 6 hours before the procedure, our protocol specifies that the final sachet is to be consumed within 4 to 5 hours of colonoscopy, with completion at least 2 hours before the procedure. This adjustment reflects both local institutional practice and MOH guidance, which emphasize flexibility in timing provided the final ingestion is not less than 2 hours before sedation. Importantly, our regimen preserves the evidence-based principles underpinning ESGE guidance, namely, split dosing with a morning-of-procedure component and ensuring a minimum 2-hour fasting interval before sedation, both of which are strongly associated with improved bowel cleanliness and adenoma detection rate [24,26].

We also note that prior RCTs and meta-analyses have shown that modest variations in the exact timing within this 4- to 6-hour window do not materially reduce bowel cleanliness, as long as ingestion is completed at least 2 hours before the procedure [25,26]. Our regimen, therefore, remains consistent with high-quality evidence and preserves adherence to safety requirements.

To ensure transparency, we have clarified in the manuscript that our regimen follows the ESGE 2019 guidelines as the international benchmark, while adapting the timing slightly to align with Malaysian MOH guidance and our institution’s established sedation safety policies.

**ii) Exact manuscript changes**

**a) Methods (In Interventions Section; Control Arm (Standard Counselling) Subsection)**

“The content and dosing regimen were developed with reference to the European Society of Gastrointestinal Endoscopy (ESGE) Guideline Update 2019 on bowel preparation for colonoscopy [23] and the Malaysian Ministry of Health (MOH) Clinical Practice Guidelines on colorectal cancer screening [24]. Our regimen specifies that the final sachet should be consumed 4 to 5 hours before the procedure, completed at least 2 hours before colonoscopy. This adaptation reflects local institutional policy and MOH recommendations, while remaining aligned with ESGE’s general principles of split dosing and minimum fasting intervals. This is further corroborated by evidence that modest variations within this range do not compromise bowel cleanliness or adenoma detection [25, 26].” (**Pages 15-16, Lines 198-207**)

**iii) References** **(Citation order and numbering are as in the main manuscript)**

23. Hassan C, East J, Radaelli F, Spada C, Benamouzig R, Bisschops R, et al. Bowel preparation for colonoscopy: ESGE Guideline - Update 2019. Endoscopy. 2019;51(8):775-794. doi:10.1055/a-0959-0505.

24. Ministry of Health Malaysia. Clinical Practice Guidelines: Management of Colorectal Carcinoma (including screening and prevention). 3rd ed. Putrajaya: MOH; 2017.

25. Bucci C, Rotondano G, Hassan C, Rea M, Bianco MA, Cipolletta L. Optimal bowel cleansing for colonoscopy: split the dose! A series of meta-analyses of controlled studies. Gastrointest Endosc. 2014;80(4):566-576. doi:10.1016/j.gie.2014.04.012.

26. Martel M, Barkun AN, Menard C, Restellini S, Kherad O, Vanasse A. Split-dose preparations are superior to day-before bowel cleansing regimens: a meta-analysis. Gastroenterology. 2015;149(1):79-88. doi:10.1053/j.gastro.2015.04.004.

___________________________________________________________________________

**4) Reviewer’s Major Comment 4: Version Control and Stability of the Chatbot (ChatGPT) Intervention**

Given that your intervention relies on a proprietary OpenAI model that is periodically updated, how will you ensure that participants receive consistent guidance throughout the trial?

**i) Author’s Response**

We thank the reviewer for raising this important concern about the consistency of an externally developed AI system. We fully agree that reproducibility could be compromised if trial participants were exposed to model drift due to periodic updates from OpenAI. To address this, we do not rely on live access to the model. Instead, we are using a **locked local deployment with fixed prompts and templated outputs** that was finalized prior to trial commencement.

The chatbot’s core educational content, covering diet restrictions, purgative timing, fasting intervals, and procedural instructions, is implemented as templated, pre-specified text blocks. These remain identical across all participants and are not subject to spontaneous regeneration or changes. The underlying large language model is used only to deliver these templates in natural, conversational language and to provide clarification when patients pose free-text questions. For transparency and quality assurance, all interactions are stored as transcripts with date and time stamps and will be monitored periodically by the trial’s personnel.

This layered approach-frozen content, templated messaging, and transcript logging ensures that all participants receive the same evidence-based guidance throughout the trial, independent of external updates to the underlying OpenAI system.

**ii) Exact manuscript changes**

**Methods: In Intervention Arm (AI-ChatGPT Chatbot Counseling):**

“To ensure the participants received consistent guidance throughout the trial, the ChatGPT used in this trial runs on a locked local deployment finalized before the trial starts to eliminate variability due to external model updates. Core educational content, including dietary restrictions, purgative timing, and fasting intervals, is implemented as templated outputs to ensure that all participants receive identical evidence-based instructions. The language model delivers this content conversationally and provides clarifications, but does not alter the dosing regimen. All interactions are stored as transcripts with date and time stamps for monitoring.” (**Pages 17, Lines 230-237**)

___________________________________________________________________________

**5) Reviewer’s Major Comment 5: Risk of Inaccurate Advice Due to High Chatbot Temperature Setting**

The protocol states that patients will interact directly with the ChatGPT-based chatbot without real-time expert oversight and that the model will operate with a temperature parameter of 1.0. This raises concern that high temperature may increase ambiguous or inaccurate advice.

**i) Author’s Response**

We thank the reviewer for raising this important point. We recognize that higher temperature values can increase variability in generative text, potentially leading to ambiguous outputs. To mitigate this, our design uses several safeguards.

First, all dosing and safety-critical content (including diet restrictions, purgative timing, fasting intervals, and sedation precautions) is delivered as fixed, templated messages that are identical for every participant. The model is used only to present these templates in conversational form and to answer clarification questions. Thus, the temperature parameter does not influence regimen instructions.

Second, we selected a temperature of 1.0 only after pilot testing showed that lower values (0-0.7) produced rigid, sometimes confusing phrasing that reduced patient comprehension, while 1.0 maintained natural dialogue without compromising accuracy.

Third, all interactions are automatically logged, and transcripts are reviewed by the trial safety officer under the pre-specified error taxonomy. Any deviation from templated guidance is escalated, with immediate suspension in the event of a critical error. Patients are also instructed to seek staff assistance if any answer is unclear, with direct human counselling available as a safety net.

Together, these measures ensure that the chosen temperature parameter enhances engagement without introducing risk.

**ii) Exact manuscript changes**

**a) Methods: In Intervention Arm (AI-ChatGPT Chatbot Counseling)**

“A temperature parameter of 1.0 was chosen since our pilot testing showed that lower values (0.0 - 0.7) produced rigid, sometimes confusing phrasing that reduced patient comprehension, while 1.0 maintained natural dialogue without compromising accuracy of instruction for bowel preparation. Besides, all dosing and safety-critical instructions (dietary restrictions, purgative timing, fasting intervals, sedation precautions) are delivered using templated, pre-specified messages to ensure consistency across participants. The temperature parameter affects only conversational phrasing and clarifications, selected to improve patient comprehension and engagement.” (**Page 16, Lines 221-229**)

**b) Methods: In the whole section of AI Error Taxonomy, Threshold and Escalation**

“All chatbot interactions are logged and monitored under the pre-specified error taxonomy. All chatbot interactions will be logged and reviewed according to a pre-specified error taxonomy. Errors are classified as Critical, Major or Minor (definitions below). A weekly random sample of 10% of sessions will be reviewed by trained clinicians and review sampling will expand to 25% if thresholds are exceeded. Escalation rules are:

1. Critical error: any instance of advice that could cause direct physical harm (e.g., wrong dosing interval for the laxatives leading to electrolyte imbalance; instruction to discontinue essential medication without context) will immediately suspend the affected module and trigger urgent review by the Safety Officer and Data Monitoring Committee (DMC).

2. Major errors: defined as incorrect or misleading clinical advice that could materially reduce bowel preparation adequacy (e.g., wrong laxative timing exceeding 6 hours). If 1% or more of audited sessions in a rolling 2-week window contain Major errors, root cause analysis will be performed, chatbot prompts/content will be corrected, and audit sampling will increase to 25% until the error rate falls below the threshold.

3. Minor errors: non-material issues such as ambiguous wording, style, or formatting. If more than 5% of audited sessions contain minor errors in two consecutive reviews, content will be refined and monitored more closely.

This structured governance approach aligns with international AI safety recommendations, including WHO and SPIRIT-AI/CONSORT-AI guidance [29, 30], healthcare AI safety principles [31-33], the National Institute of Standards and Technology (NIST) AI Risk Management Framework [34], and explainability principles for multidisciplinary evaluation of AI in health [35].” (**Page 19-20, Lines 285-307**)

**iii) References** **(Citation order and numbering are as in the main manuscript)**

29.	World Health Organization. Ethics and governance of artificial intelligence for health: WHO guidance. Geneva: World Health Organization; 2021. Available from: https://iris.who.int/bitstream/handle/10665/341996/9789240029200-eng.pdf?sequence=1. Date of access: 27th May 2025.

30.	Ibrahim H, Liu X, Rivera SC, Moher D, Chan AW, Sydes MR, Calvert MJ, Denniston AK. Reporting guidelines for clinical trials of artificial intelligence interventions: the SPIRIT-AI and CONSORT-AI guidelines. Trials. 2021;22(1):11. doi: 10.1186/s13063-020-04951-6.

31.	Coiera EW, Verspoor K, Hansen DP. We need to chat about artificial intelligence. Med J Aust. 2023;219(3):98-100. doi: 10.5694/mja2.51992.

32.	Topol E. Preparing the healthcare workforce to deliver the digital future: The Topol Review. An independent report on behalf of the Secretary of State for Health and Social Care. London (UK): Health Education England; 2019. Available from: https://topol.hee.nhs.uk/wp-content/uploads/HEE-Topol-Review-2019.pdf. Date of access: 3rd Sep 2025.

33.	Joshi I, Morley J. Artificial Intelligence: How to get it right. Putting policy into practice for safe data-driven innovation in health and care. London (UK): NHSX; 2019:1-55. https://www.nhsx.nhs.uk/ai-lab/explore-all-resources/understand-ai/artificial-intelligence-how-get-it-right. Date of access: 3rd September 2025.

34.	National Institute of Standards and Technology (NIST). Artificial Intelligence Risk Management Framework (AI RMF 1.0). NIST AI 100-1. Gaithersburg (MD): U.S. Department of Commerce; 2023. doi: 10.6028/NIST.AI.100-1.

35.	Amann J, Blasimme A, Vayena E, Frey D, Madai VI. Explainability for artificial intelligence in healthcare: a multidisciplinary perspective. BMC Med Inform Decis Mak. 2020;20(1):310. doi: 10.1186/s12911-020-01332-6.

___________________________________________________________________________

**6) Reviewer’s Major Comment 6:** **Lack of Defined Threshold for Acceptable Error**

The protocol does not define what level of error would be considered unacceptable.

**i) Author’s Response:**

We thank the reviewer for emphasizing this critical point. We agree that pre-specifying error thresholds is essential for transparent governance of an AI-delivered intervention. In the revised protocol we now define an error taxonomy (Critical, Major, Minor) and escalation rules, so that both investigators and the Data Monitoring Committee can respond consistently to any unexpected behavior.

- **Critical errors** are outputs that could cause direct harm if followed (for example, incorrect dosing interval risking electrolyte imbalance, or erroneous instructions about fasting before sedation). Any single Critical error leads to immediate suspension of the affected chatbot pathway and urgent review by the trial Safety Officer and Data Monitoring Committee.
- **Major errors** are incorrect but non-life-threatening outputs that could meaningfully reduce bowel-prep adequacy (for example, instructing the final laxative dose more than 12 hours before colonoscopy). A rate of ≥1% of audited transcripts within a rolling 2-week window triggers corrective action, including prompt content adjustment and expanded audit sampling.
- **Minor errors** are typographical or stylistic issues without clinical impact. More than 5% minor error prevalence on two consecutive audits triggers content polishing and monitoring.

All chatbot transcripts are logged with time-stamps and metadata. Ten percent are reviewed weekly by trained clinicians, and this expands to 25% whenever a threshold breach occurs. Summary reports are provided monthly to the Data Monitoring Committee. This framework ensures that error handling is predefined, risk-based, and fully auditable, aligning with best-practice recommendations for AI safety in clinical trials [1–3].

**ii) Exact manuscript changes**

**a) Methods: In the whole section of AI Error Taxonomy, Threshold and Escalation**

“All chatbot interactions are logged and monitored under the pre-specified error taxonomy. All chatbot interactions will be logged and reviewed according to a pre-specified error taxonomy. Errors are classified as Critical, Major or Minor (definitions below). A weekly random sample of 10% of sessions will be reviewed by trained clinicians and review sampling will expand to 25% if thresholds are exceeded. Escalation rules are:

1. Critical error: any instance of advice that could cause direct physical harm (e.g., wrong dosing interval for the laxatives leading to electrolyte imbalance; instruction to discontinue essential medication without context) will immediately suspend the affected module and trigger urgent review by the Safety Officer and Data Monitoring Committee (DMC).

2. Major errors: defined as incorrect or misleading clinical advice that could materially reduce bowel preparation adequacy (e.g., wrong laxative timing exceeding 6 hours). If 1% or more of audited sessions in a rolling 2-week window contain Major errors, root cause analysis will be performed, chatbot prompts/content will be corrected, and audit sampling will increase to 25% until the error rate falls below the threshold.

3. Minor errors: non-material issues such as ambiguous wording, style, or formatting. If more than 5% of audited sessions contain minor errors in two consecutive reviews, content will be refined and monitored more closely.

This structured governance approach aligns with international AI safety recommendations, including WHO and SPIRIT-AI/CONSORT-AI guidance [29, 30], healthcare AI safety principles [31-33], the National Institute of Standards and Technology (NIST) AI Risk Management Framework [34], and explainability principles for multidisciplinary evaluation of AI in health [35].” **(Page 19-20, Lines 285-307)**

**iii) References (Citation order and numbering are as in the main manuscript)**

29. World Health Organization. Ethics and governance of artificial intelligence for health: WHO guidance. Geneva: World Health Organization; 2021. Available from: https://iris.who.int/bitstream/handle/10665/341996/9789240029200-eng.pdf?sequence=1. Date of access: 27th May 2025.

30. Ibrahim H, Liu X, Rivera SC, Moher D, Chan AW, Sydes MR, Calvert MJ, Denniston AK. Reporting guidelines for clinical trials of artificial intelligence interventions: the SPIRIT-AI and CONSORT-AI guidelines. Trials. 2021;22(1):11. doi: 10.1186/s13063-020-04951-6.

31. Coiera EW, Verspoor K, Hansen DP. We need to chat about artificial intelligence. Med J Aust. 2023;219(3):98-100. doi: 10.5694/mja2.51992.

32. Topol E. Preparing the healthcare workforce to deliver the digital future: The Topol Review. An independent report on behalf of the Secretary of State for Health and Social Care. London (UK): Health Education England; 2019. Available from: https://topol.hee.nhs.uk/wp-content/uploads/HEE-Topol-Review-2019.pdf. Date of access: 3rd Sep 2025.

33. Joshi I, Morley J. Artificial Intelligence: How to get it right. Putting policy into practice for safe data-driven innovation in health and care. London (UK): NHSX; 2019:1-55. https://www.nhsx.nhs.uk/ai-lab/explore-all-resources/understand-ai/artificial-intelligence-how-get-it-right. Date of access: 3rd September 2025.

34. National Institute of Standards and Technology (NIST). Artificial Intelligence Risk Management Framework (AI RMF 1.0). NIST AI 100-1. Gaithersburg (MD): U.S. Department of Commerce; 2023. doi: 10.6028/NIST.AI.100-1.

35. Amann J, Blasimme A, Vayena E, Frey D, Madai VI. Explainability for artificial intelligence in healthcare: a multidisciplinary perspective. BMC Med Inform Decis Mak. 2020;20(1):310. doi: 10.1186/s12911-020-01332-6.

___________________________________________________________________________

**7) Reviewer’s Major Comment 7: Impact of Eligibility Criteria on Study Population with Potential Bias from Language and Digital Literacy Requirements**

Your eligibility criteria favour participants with “basic digital literacy” and fluency in English or Malay… could the authors comment on the selection bias?

**i) Author’s Response**

We thank the reviewer for highlighting this concern. We acknowledge that requiring basic digital literacy and fluency in English or Malay limits generalizability, particularly for older adults and those at higher risk of poor bowel preparation. This restriction was a deliberate choice for a first-in-context safety and feasibility trial. Early-phase digital and AI health interventions typically begin with populations able to interact reliably with the technology, in order to establish internal validity and content safety before extending to groups with greater access barriers [1-3].

To mitigate this limitation, we provided loaner tablets and a short onboarding session (≤10 minutes) for participants lacking personal devices but otherwise eligible. We also record baseline digital literacy and language fluency in the case report form. These variables will be reported descriptively in the results, so the reader can judge the representativeness of the trial cohort. We emphasize that we do not plan inferential subgroup analyses for digital literacy strata, given the modest sample size, but will transparently report these distributions. Future implementation studies will extend inclusivity through simplified interfaces, voice-based interactions, and multilingual adaptations.

**ii) Exact manuscript changes**

**a) In Methods (Subsection Eligibility Criteria**)

“We require participants to have basic digital literacy in order to interact with the chatbot interface. For participants who are otherwise eligible but lack a personal device, the trial will provide a loaner tablet and a short onboarding session (10 minutes or less) to enable participation. The provision of loaner devices aims to mitigate but not eliminate selection bias; the trial remains a pragmatic first-phase evaluation among individuals who can use a mobile/tablet interface.” (**Page 13; Lines 149-155**)

**b) In Discussion (Subsection Study Limitations**)

Second, requiring basic digital literacy and fluency in English or Malay may limit generalizability of our study findings. This first-phase trial intentionally restricts inclusion to participants able to engage with the intervention so that safety and fidelity of digital health-based intervention administration can be established without technical confounders [67-69]. Future work will adapt the system for broader populations (multilingual versions, voice interfaces, assisted navigation) and test effectiveness in individuals with limited digital or health literacy.” (**Pages 36 - 37; Lines 717** - **723**)

**iii) References (Citation order is as in the main manuscript)**

67. Marcolino MS, Oliveira JAQ, D'Agostino M, Ribeiro AL, Alkmim MBM, Novillo-Ortiz D. The Impact of mHealth Interventions: Systematic Review of Systematic Reviews. JMIR Mhealth Uhealth. 2018;6(1):e23. doi: 10.2196/mhealth.8873.

68.	Mohr DC, Weingardt KR, Reddy M, Schueller SM. Three Problems With Current Digital Mental Health Research . . . and Three Things We Can Do About Them. Psychiatr Serv. 2017;68(5):427-429. doi: 10.1176/appi.ps.201600541.

69. Whittaker R, Merry S, Dorey E, Maddison R. A development and evaluation process for mHealth interventions: examples from New Zealand. J Health Commun. 2012;17 Suppl 1:11-21. doi: 10.1080/10810730.2011.649103.

___________________________________________________________________________

**8) Reviewer Minor Comment 1:** “Dr (Surgery)” for Nabil Mohammad Azmi: This is an uncommon degree abbreviation. Please check.

**i) Author’s Response:**

We thank the reviewer for noting this. We confirm that “Dr (Surgery)” is the official Universiti Kebangsaan Malaysia (UKM) designation for *Doctor of Surgery*, which is the successor to the research-based *Master of Surgery* degree. As this is the formal institutional usage, no change has been made.

___________________________________________________________________________

**9) Reviewer Minor Comment 2:** Line 203: “No PII…..” is at first use. Line 244 “PII” has a full term. The abbreviation should be defined upon its first use.

**i) Author’s Response:**

We agree with the reviewer. The term has now been revised to “Personally Identifiable Information (PII)” upon first use at Line 203, with the acronym applied consistently thereafter.

___________________________________________________________________________

**10) Reviewer’s Minor Comment 3:** Line 220: “will be assessed using the ‘Flesh Kincaid’ Grade Level score” should be “Flesch Kincaid.”

**i) Author’s Response:**

We thank the reviewer for identifying this typographical error. The manuscript has been corrected to “Flesch Kincaid Grade Level score.”

___________________________________________________________________________

**11) Reviewer’s Minor Comment 4:** Line 395: should be “TMG.”

**i) Author’s Response:**

We acknowledge the correction and have amended the manuscript accordingly to read “TMG.”

___________________________________________________________________________

**12) Reviewer’s Minor Comment 5:** The manuscript uses both “counselling” and “counseling.” Please review the text for consistency and adopt a single preferred form throughout.

**i) Author’s Response:**

We thank the reviewer for highlighting this inconsistency. The manuscript has been reviewed and revised for consistency, adopting “counselling” throughout, in line with British English usage.

___________________________________________________________________________

**13) Reviewer’s Minor Comment 6:** Line 535–536: “The primary outcome, the BBPS, is a validated measure of bowel cleanliness [18].” Reference [18] is for a psychiatric study using Woebot (*JMIR Ment Health*. 2017). The correct reference for BBPS validation would be Lai EJ et al. (*Gastrointest Endosc*. 2009).

**i) Author’s Response:**

We agree with the reviewer. The previous reference was cited in error. It has been corrected to Lai EJ, Calderwood AH, Doros G, Fix OK, Jacobson BC. The Boston Bowel Preparation Scale: a valid and reliable instrument for colonoscopy-oriented research. Gastrointest Endosc. 2009;69(3 Pt 2):620-625, which is the validated source for BBPS.

___________________________________________________________________________

**14) Reviewer’s Minor Comment 7:** Reference 7 and 10 appear to be duplicates.

**i) Author’s Response:**

We thank the reviewer for this observation. The references have been carefully reviewed and reference 10 has been replaced with this new reference: Jiang XH, Yuan XH, Zhao H, Peng JS. The Effectiveness and Feasibility of Conversational Agents in Supporting Care for Patients With Cancer: Systematic Review and Meta-Analysis. J Med Internet Res. 2025 Aug 8;27:e76968.
